# The effect of oral administration of undenatured type II collagen on monosodium iodoacetate-induced osteoarthritis in young and old rats

**DOI:** 10.1038/s41598-023-33763-2

**Published:** 2023-04-20

**Authors:** Emre Sahin, Cemal Orhan, Fusun Erten, Zainulabedin Saiyed, Elnaz Karimian Azari, Shane Durkee, Kazim Sahin

**Affiliations:** 1 Department of Animal Nutrition, Faculty of Veterinary Medicine, Bingol University, Bingol, 12100 Turkey; 2 Department of Animal Nutrition, Faculty of Veterinary Medicine, Firat University, Elazig, 23119 Turkey; 3 Department of Veterinary Science, Pertek Sakine Genc Vocational School, Munzur University, Tunceli, 62500 Turkey; 4 Lonza Greenwood LLC, Greenwood, SC 29646 USA

**Keywords:** Diseases, Medical research

## Abstract

We investigated whether different doses of undenatured type II collagen (undenatured collagen, UC-II) help improve monosodium iodoacetate (MIA)-induced (osteoarthritis) OA in young and old rats. A total of 70 rats were divided into five groups: (1) control; (2) MIA (a single intra-articular injection of MIA); (3)–(5) MIA+ Undenatured Collagen with various oral doses (0.66, 1.33, and 2 mg/kg). The results showed that all doses of undenatured collagen in both age groups reduced knee diameter, while the two higher doses (1.33 mg/kg and 2 mg/kg) reduced the Mankin score and increased most gait measurements as early as day 14 compared to the MIA rats. However, the 2 mg/kg dose showed the best efficacy in improving Mankin score and gait measurements by 28 days post-OA induction. In young but not old rats, all doses of undenatured collagen reduced the Kellgren-Lawrence score compared to the MIA group. Undenatured collagen reduced the levels of most inflammatory and cartilage breakdown markers in serum and knee joint cartilage in both age groups. In conclusion, this data suggests that while all doses of undenatured collagen supplementation may ameliorate MIA-induced OA symptoms, the higher doses showed faster improvement in gait measurements and were more efficacious for overall joint health in rats.

## Introduction

Osteoarthritis (OA) affects over 500 million people worldwide and is considered one of the main reasons for disability. Globally, the proportion of people affected by OA has notably increased by 48% from 1990 to 2019^1^. While many assume that OA is most prevalent in older individuals, previous studies have shown that more than half of patients diagnosed with symptomatic knee OA are under the age of 65^2^. The increasing number of sports injuries, joint trauma, occupational exposure, and obesity have resulted in an increase in OA incidence among younger individuals^3, 4^. Inflammatory arthritis, which comprises inflammatory cell recruitment, synovial joint inflammation, and articular cartilage degeneration, is an age-related joint disease^5^. In parallel with the aging process, the systemic inflammatory status gradually intensifies due to an increase in age-related inflammation markers; thus, the risk of OA progression increases in older individuals^6^. Excessive activation of the nuclear factor kappa B (NF-κB) pathway may stimulate the production of inflammatory cytokines [such as interleukin 1-β (IL-1β), interleukin 6 (IL-6), and tumor necrosis factor α (TNF-α)] in chondrocytes, leading to the destruction of the joint structure and the onset of OA^7^. IL-1 plays a central role in immune disorders by activating intracellular T-cell interactions. IL-1β and TNFα are essential triggers of OA development, whereas IL-4, transforming growth factor β (TGF-β), and interferon-gamma (IFNγ) are categorized as anti-inflammatory cytokines in OA^8^. The increase in inflammatory cytokines and reactive oxygen species (ROS) with aging also stimulate matrix metalloproteinases (MMPs) that drive cartilage degradation in OA^9, 10^. Additionally, gut microbiota dysbiosis may contribute to OA progression due to a chronic systemic inflammatory process that may lead to cartilage degeneration^11^. Aging also exacerbates intestinal dysbiosis, increases proinflammatory symbionts, and reduces antigen-presenting T-cell activation^12^. It has been shown previously that modulating gut microbiota with dietary supplements, such as chondroitin sulfate and probiotics can alleviate OA symptoms in OA-induced rats^11^. In addition to aging-related factors, mechanical factors may also trigger the formation of OA earlier in life. Overloading the joint may stimulate ROS formation and inhibit proteoglycan production. This process exacerbates inflammatory cytokine production^13^ and chondrocyte senescence, similar to aging^14^. The MIA-induced OA model, extensively used in experimental studies, mimics these phases of OA progression, including initial inflammatory and pain behavior in young and old rats^15–17^.

UC-II undenatured type II collagen (undenatured collagen) is a patented form of collagen derived from chicken sternum cartilage^18^. Undenatured collagen provides > 3% undenatured (native) type II collagen for joint health support, and is produced using a gentle process designed to retain its native triple helix structure^19^ and preserve its biological activity. A small amount of undenatured collagen (40 mg/day) has been shown to induce a process known as oral tolerance that engages the immune system in repairing joint cartilage and reducing arthritis symptoms^20^. Undenatured collagen preserves glycosylated antigenic regions, which are recognized by the T-cells^18^. When undenatured collagen is administered orally, it interacts with Peyer’s patches in the gut and activates immune cells, transforming naive T-cells into T regulatory (Treg) cells that specifically recognize type II collagen^18, 20, 21^. Although many animal^22–25^ and human^18, 26, 27^ studies have demonstrated the beneficial effect and possible molecular mechanism of low-dose undenatured collagen administration on OA, the dose–response effect of undenatured collagen in different age groups is still unclear. Understanding the effect of different doses of undenatured collagen on OA may contribute to treatment recommendations for age- or injury-related OA in humans and provide supportive information for animals prone to joint disorders.

The data on the use of different doses of undenatured collagen in the treatment of OA is limited. It has been reported that different amounts of undenatured collagen may have differing pharmacological effects, especially on oral tolerance mechanisms^19, 28^. It has previously been shown that high doses of undenatured collagen (daily 480 and 640 mg) are more effective than lower doses (daily 80 mg) in eliminating clinical arthritis symptoms in horses^29^. However, these studies lacked systemic and molecular evaluation of various low doses of undenatured collagen within different age groups for OA. Therefore, we aimed to compare the efficacy of different doses of undenatured collagen in MIA-induced knee OA in young and old rats.

## Materials and methods

### Animals

All procedures were conducted in compliance with the ARRIVE guidelinesThey were conducted in strict compliance with the relevant laws, the Animal Welfare Act, the Public Health Services Policy, and guidelines established by the Institutional Animal Care and Use Committee of the Institute and the European Union guidelines (No. 2010/63/EU) for the protection of the laboratory animals used for scientific purposes. The experimental procedures were approved by the Animal Experiments Local Ethics Committee of Bingol University (20-04/01-02). All efforts were made to minimize animal suffering and the number of animals used. Rats were maintained under standard environmental conditions (a temperature of 22 ± 2 °C, 12 h light-12 h dark cycle, and 55% ± 3% humidity) in propylene cages (47 × 37 × 19 cm, three or four rats per cage) with ad libitum water and food.

A total of 70 female Wistar albino rats (35 young rats, eight weeks old, weighing 170 ± 20 g; 35 old rats, eighteen months old, weighing 340 ± 20 g) were obtained from the Bingol University Experimental Research Center. We chose female rats for this model because humoral and cellular immune responses to antigenic stimulation are more pronounced in females, and testosterone has an anti-inflammatory effect on arthritis. Therefore, the susceptibility of female rats to experimental arthritis is higher than male rats^30^.

### Experimental design

**Figure 1:**
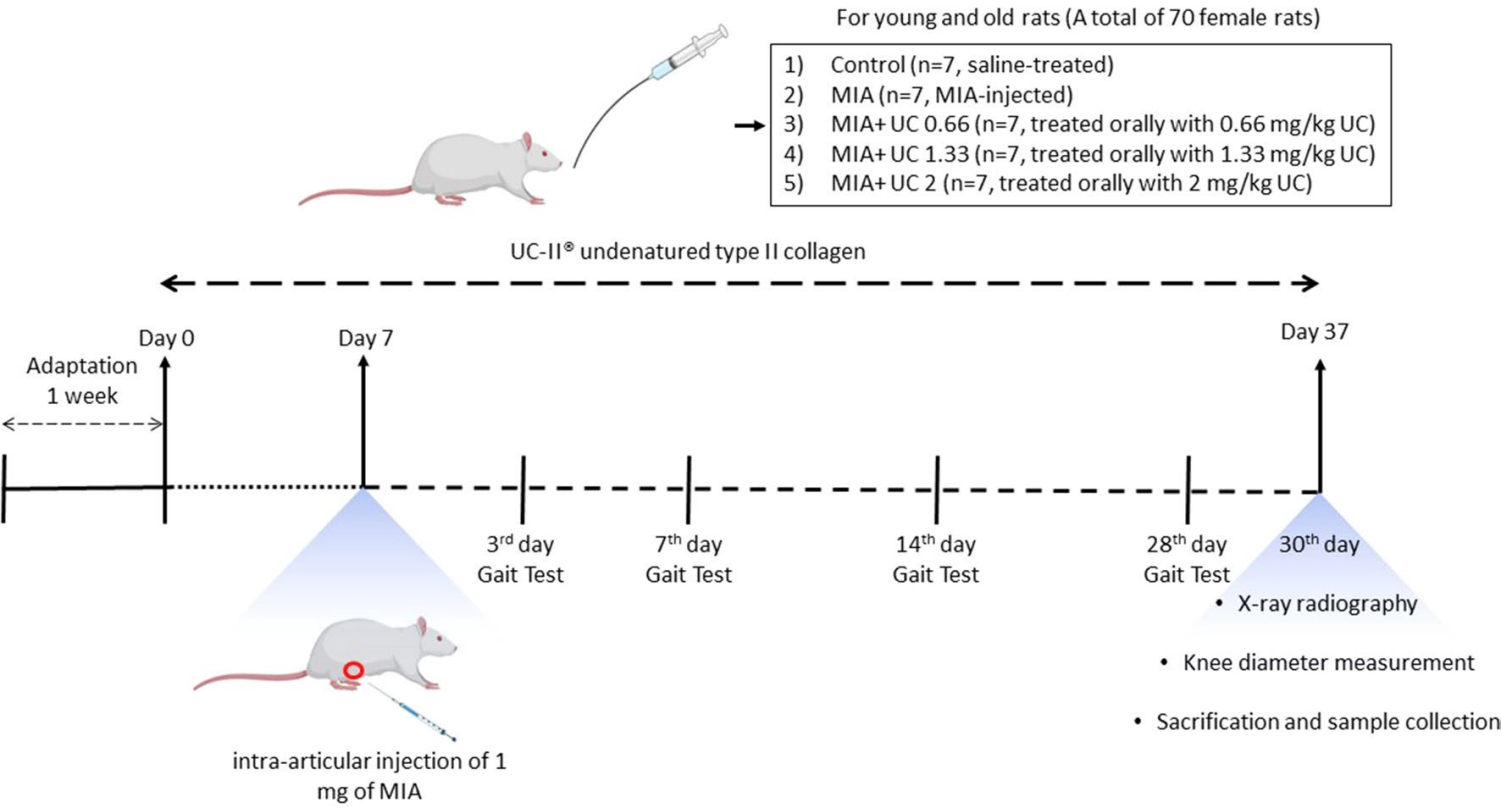
The experimental design of the study. MIA, monosodium iodoacetate; UC, undenatured collagen.

After a week of acclimatization period, both young and old animals were divided into five groups, with seven animals in each group (Fig. 1): (1) control (saline-treated); (2) MIA (a single intra-articular injection of MIA); (3) MIA + UC 0.66 (0.66 mg/kg undenatured collagen-treated); (4) MIA + UC 1.33 (1.33 mg/kg undenatured collagen-treated); (5) MIA + UC 2 (2 mg/kg undenatured collagen-treated). Undenatured collagen doses were determined based on the human equivalent dose (HED) used in previous clinical studies^31^. HED doses (40, 80, and 120 mg/day) were converted to rat doses (0.66, 1.33, and 2 mg/kg), according to Shin et al.^32^. The UC-II undenatured type II collagen was obtained from Lonza Greenwood LLC. It is a powdered, glycosylated, and shelf-resistant component (Batch no:20GW246506). The product contains > 3% undenaturated type 2 collagen and > 25% total collagen. Normal saline (1 mL) was used as a vehicle for three undenatured collagen doses. Also, MIA rats received 1 mL of normal saline daily. Rats were dosed once daily with undenatured collagen by gastric gavage for 37 days, and OA was induced on the seventh day after initiation of undenatured collagen supplementation. Initially, rats were anesthetized using xylazine (10 mg/kg) and ketamine hydrochloride (50 mg/kg). Then the right knee of all rats was shaved for injection and disinfected with 70% alcohol. 1 mg of MIA (Sigma, St. Louis, USA) was dissolved in 50 μL sterile saline and injected into the right knee joint cavity through the infrapatellar ligament with a 29-G needle to induce knee OA^33^. In addition, 50 μL saline was injected into the right knee joint of the control rats.

At the end of the undenatured collagen supplementation period (37th day), rats were weighed and sacrificed by decapitation under anesthesia (10 mg/kg xylazine and 50 mg/kg ketamine hydrochloride). Blood and articular cartilage samples were quickly removed. The right knee joints of animals were removed from the muscle tissue. Blood samples were centrifuged (2000 g for 10 min), and serum samples were collected into microtubes. Serum and articular cartilage samples were stored at − 80 °C for further analysis.

### Gait test

The ink print method was used for the gait test^23^. The hind paws of each rat were dipped in black ink briefly, and then rats walked on a sheet of white paper (60 cm × 7 cm). The gait test was performed for each rat on the 3rd, 7th, 14th, and 28th days after the MIA injection. The white paper was scanned and transferred to a computer. Paw area (cm^2^), stride length (cm), and pad width (cm) were measured with Image J software (National Institutes of Health, USA). The size of the area around the right hind paw was defined as the paw area (cm^2^), the distance of the same right hind paw between two steps as stride length (cm), and the distance between the first and fifth toes of the right hind paw as pad width (cm). The arithmetic means of at least three measurements of paw areas, stride length, and pad width were considered for each rat.

### Radiographic assessment and knee diameter measurement

For assessing the knee joint osteoarthritic deterioration, X-ray radiography of each animal’s right knee joint was taken immediately after dissection (37th day), and the Kellgren-Lawrence scoring system (grade 0: none, grade 1: doubtful, grade 2: minimal, grade 3: moderate, and grade 4: severe joint deformation) was used for radiographic assessment^34^. Radiographs were examined by a radiologist blinded to the groups during the entire study period. The mediolateral diameter (mm) of the right knee joint was measured at least three times with a digital caliper (Mitutoyo, Kawasaki, Japan) to assess joint swelling before dissection (37th day).

### Histopathological analysis and Mankin classification

First, a portion of the right knee joint of the rats was stored in a formalin solution (10%) for several days. Next, joint cartilage samples were stored in a nitric acid solution (10%) for 30 days to decalcify the inorganic matter. Then, joint cartilage samples were blocked in paraffin and sectioned as 4-µm thick. Finally, the slides were stained with hematoxylin–eosin (H.E.), and the Mankin scoring system (Structure; score 0: smooth intact surface, score 1: slight surface irregularities, score 2: pannus/surface fibrillation, score 3: clefts into the transitional zone, score 4: clefts into the radial zone, score 5: clefts into the calcified zone, and score 6: total disorganization. Cells; score 0: uniform cell distribution, score 1: diffuse cell proliferation, score 2: cell clustering, and score 3: cell loss. Tidemark integrity; score 0: intact and score 1: vascularity) was used for the histopathological assessment of knee joint cartilage^35^. Tissue sections were assessed under a light microscope (LM) for the pathological features of LM by an experienced histopathologist blinded to the study groups during the entire study period.

### Analyses of serum markers

A microplate reader (Elx-800, Bio-Tek Instruments Inc, Vermont, USA) was used to determine serum levels of IL-1β (catalog no: MBS2023030, intra- and inter CV was < 10% and < 12%), IL-6 (catalog no: MBS2701082, intra- and inter CV was < 10% and < 12%), TNF-α (catalog no: MBS2088120, intra- and inter CV was < 10% and < 12%), cartilage oligomeric matrix protein (COMP, catalog no: MBS267386, intra- and inter CV was < 8% and < 12%), c-reactive protein (CRP, catalog no: MBS2702539, intra- and inter CV was < 10% and < 12%), and prostaglandin E2 (PGE2, catalog no: MBS262150, intra- and inter CV was < 8% and < 12%) with rat specific enzyme-linked immunosorbent assay (ELISA) kits according to the manufacturer's procedure (MyBioSource, Inc. San Diego, CA, USA). The lower limit of variation of IL-1β, IL-6, TNF-α, COMP, CRP, and PGE2 was 15.6 pg/mL, 7.8 pg/mL, 15.6 pg/ml, 3.12 ng/mL, 0.78 ng/mL, and 15.6 pg/mL, respectively.

### Western blot analysis

The levels of IL-1β, IL-6, IL-10, TNF-α, cyclooxygenase-2 (COX2), NF-κB, collagen type 2, matrix metalloproteinase-3 (MMP3), COMP, and TGF-β in joint cartilage were determined by Western Blot method^23^. Joint cartilage tissues of each group were pooled and homogenized with a lysis buffer containing a protease inhibitor cocktail. The protein density of homogenates was measured by Qubit 2.0 Fluorometer (Invitrogen, Life Technologies Corporation, Carlsbad, CA, USA). A total of 20 µg of protein for each group was separated by sodium dodecyl sulfate–polyacrylamide gel electrophoresis (SDS-PAGE) and then transferred into a 0.45 µm nitrocellulose membrane (GVS, Bologna, Italy). The membrane was blocked for 1 h with 5% bovine serum albumin. Next, the membranes were incubated overnight at 4 °C with diluted (1:1000) primary antibodies (Santa Cruz Bio., IL-1β; sc-52012, IL-6; sc-57315, IL-10; sc-365858, TNF-α; sc-52746, NF- κB; sc-8008, MMP3; sc-21732, COMP; sc-374660, and Abcam COX2; ab283593, collagen type 2; ab34712, TGF-β; ab215715). Then, the membranes were treated with goat anti-mouse (Santa Cruz Bio., sc-2005) and goat anti-rabbit secondary antibodies (Abcam, ab205718). Diaminobenzidine substrate was used to visualize the interaction between primary and secondary antibodies. Bands were analyzed densitometrically using the Image J software (National Institute of Health, Bethesda, USA). β-actin (Sigma Aldrich, MA1-140) was used to check protein loading. Blots were repeated three times to confirm the reliability of the results.

### Statistical analysis

A power analysis was performed using G*Power (Version 3.1.9.4) software to determine the sample size. For both age groups, the sample size was computed as seven rats per treatment group (0.05 type I error, 0.8 effect size, and a power of 90%). Data were presented as the mean ± standard error of the mean or box‐and‐whisker plots (with min and max values). SPSS software version 22.0 (IBM Corp., Armonk, NY, USA) was used for all statistical analyses. Shapiro–Wilk and Levene's tests were performed to analyze the normality of the data and the homogeneity of the variances, respectively. One-way ANOVA and Tukey post hoc tests were used for the parametric data, while the Kruskal–Wallis and Mann–Whitney tests were used for non-parametric data. p < 0.05 level was considered statistically significant.

### Ethical statement

The experimental procedures were approved by The Animal Experiments Local Ethics Committee of Bingol University (2020/04-04/01-02). The animal experiment was designed and reported according to the ARRIVE guidelines.

## Results

### Gait test

**Figure 2:**
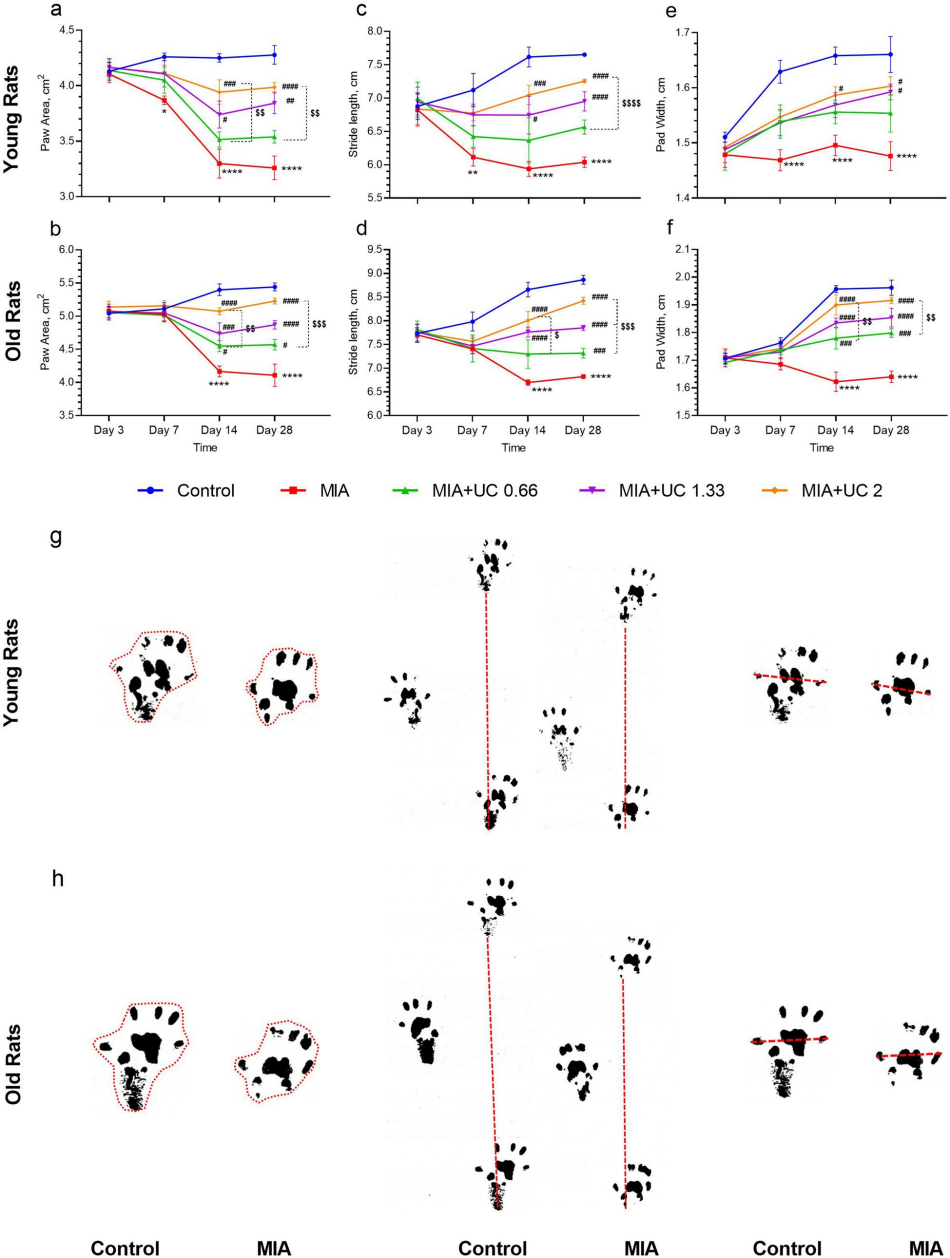
Effects of undenatured collagen (UC) supplementation at different doses on paw area (**a** for young rats, **b** for old rats), stride length (**c** for young rats, **d** for old rats), and pad width (**e** for young rats, **f** for old rats) in monosodium iodoacetate (MIA) induced osteoarthritis in young (n = 7) and old rats (n = 7). Representative images of the stride length, paw area, and pad width (**g** for young rats and **h** for old rats) measured on days 3, 7, 14, and 28 after the MIA-injection are shown. The depicted time points and error lines represent the mean ± standard error of the mean (SEM) for each time point. ANOVA and Tukey’s post hoc test were used to compare the results among different treatment groups. Statistical analysis was performed separately within each age group. Statistical significance between groups is shown by: *p < 0.05; **p < 0.01; ****p < 0.0001 (MIA vs. Control), # p < 0.05; ## p < 0.01; ### p < 0.001; #### p < 0.0001 (MIA vs. UC groups), and $$ p < 0.01; $$$ p < 0.001; $$$$ p < 0.0001 (pairwise comparisons between UC groups).

The gait test was performed on the 3rd, 7th, 14th, and 28th day post-MIA injection. As demonstrated in Fig. 2, intra-articular MIA injection impaired gait patterns compared to healthy controls in both young and old rats (Fig. 2a–f). At day seven, the gait patterns were reduced in young rats (p < 0.05 for paw area,p < 0.01 for stride length, and p < 0.0001 for pad width), whereas no statistical difference was detected in old rats (p > 0.05). At days 14 and 28 after OA-induction, gait patterns were reduced compared to the control group in young and old rats (p < 0.0001, for all). In young and old rats, 1.33 mg/kg and 2 mg/kg of undenatured collagen supplementation increased paw area and stride length as early as 14 days post-OA induction. In addition, while the 0.66 mg/kg dose improved paw area and pad width in old rats, the younger group also showed an increase, but it was not statistically significant. At day 14 in both age groups, stride length alone did not improve with 0.66 mg/kg undenatured collagen supplementation compared to MIA rats (p > 0.05). At the same time point, supplementation of 2 mg/kg of undenatured collagen increased paw area compared to 0.66 mg/kg in young and old rats (p < 0.01). Also, stride length (p < 0.05) and pad width (p < 0.01) was markedly improved by 2 mg/kg undenatured collagen compared to 0.66 mg/kg in old rats. At day 28, all doses of undenatured collagen supplementation improved all gait measurements in old rats; however, this improvement was not statistically signficant with the lower dose (0.66 mg/kg) in young rats (p > 0.05). The 2 mg/kg undenatured collagen supplementation was more effective than the 0.66 mg/kg dose in improving gait measurements in both age groups (young rats: p < 0.01 for paw area, p < 0.0001 for stride length; old rats: p < 0.001 for paw area and stride length, p < 0.01 for pad width).

### Kellgren-Lawrence score and knee diameter

**Figure 3:**
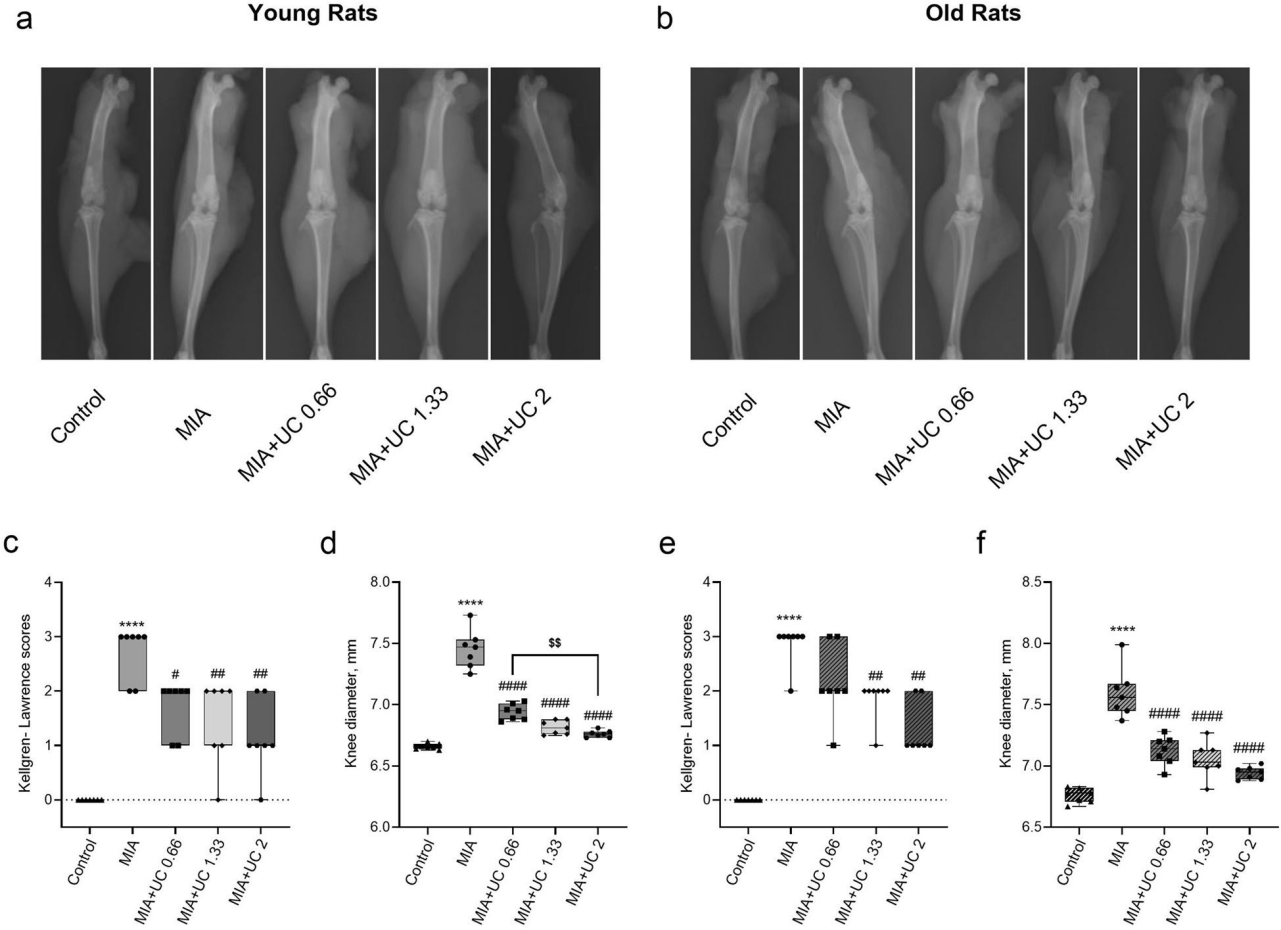
Effects of undenatured collagen (UC) supplementation at different doses on the knee joint in monosodium iodoacetate (MIA) induced osteoarthritis in young (n = 7) and old rats (n = 7). Representative radiographic images (**a** for young rats and **b** for old rats) obtained at the end of the experiment (37^th^ day) are shown. The depicted box‐and‐whisker plots represent Kellgren-Lawrence scores (**c** for young rats and **e** for old rats) and knee joint diameter (**d** for young rats and **f** for old rats) with min and max values. For Kellgren-Lawrence scores, Kruskal Wallis and Mann–Whitney U comparison test were used to compare the results among different treatment groups. ANOVA and Tukey’s post hoc test were used for joint diameter data to compare the results among different treatment groups. Statistical analysis was performed separately within each age group. Statistical significance between groups is shown by: **** p < 0.0001 (MIA vs. Control), # p < 0.05; ## p < 0.01 #### p < 0.0001 (MIA vs. UC groups), and $$ p < 0.01 (pairwise comparisons between UC groups).

At the end of the study (37th day), Kellgren-Lawrence scoring system was used to assess the severity of knee OA. MIA injection led to structural joint changes by increasing the Kellgren-Lawrence score and the joint diameter in both young and old rats, as seen in Fig. 3 (p < 0.0001). In young rats, all doses of undenatured collagen reduced the Kellgren-Lawrence score compared with MIA rats (p < 0.05 for 0.66 mg/kg and p < 0.01 for 1.33 mg/kg and 2 mg/kg). In old rats, compared with MIA rats, a significant improvement in the Kellgren-Lawrence score was achieved with the 1.66 mg/kg and 2 mg/kg doses (p < 0.01), however the lower dose of 0.66 mg/kg could not significantly reduce the score (p > 0.05).

As expected, joint diameter was higher in MIA rats compared with control rats (Fig. 3d , p < 0.0001). All doses of undenatured collagen reduced joint swelling in young and old rats compared with MIA rats (p < 0.0001). For young rats, 2 mg/kg undenatured collagen supplementation was more effective in reducing joint swelling than 0.66 mg/kg (p < 0.01). In contrast, no significant difference between undenatured collagen groups was found in older rats (p > 0.05).

### Histopathological analysis and Mankin score

**Figure 4:**
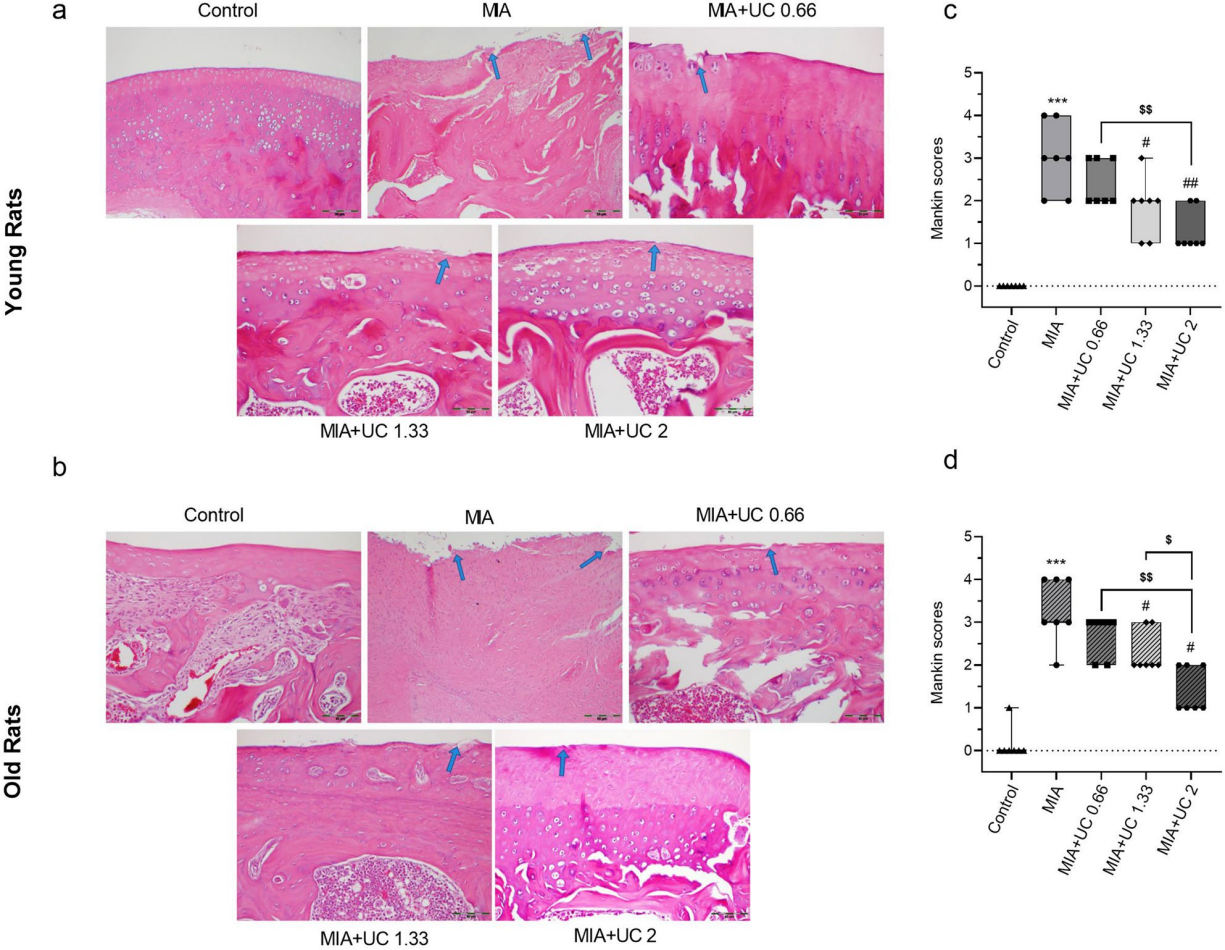
Effects of undenatured collagen (UC) supplementation at different doses on histopathology of the knee joint in monosodium iodoacetate (MIA) induced osteoarthritis in young (n = 7) and old (n = 7) rats. Representative histopathologic images of hematoxylin–eosin staining (H&E × 200, **a**: Smooth intact surface in the control group, pannus/surface fibrillation, clefs into the transitional and radial zones in the MIA group (blue arrows), mild fibrillation and clefs in MIA + UC 0.66 group (blue arrow), clefs in the MIA + UC 1.33 group (blue arrow) and slight clefs in the MIA + UC 2 were observed in young rats. (**b**): smooth intact surface in the control group, marked pannus/surface fibrillation, clefs into the transitional and radial zones in the MIA group (blue arrows), fibrillation and clefs in MIA + UC 0.66 group (blue arrow), mild fibrillation and clefs in the MIA + UC 1.33 group (blue arrow) and slight clefs in the MIA + UC 2 were observed in old rats. The depicted box‐and‐whisker plots represent Mankin scores (**c** for young rats and **d** for old rats) with min and max values. Kruskal Wallis and Mann–Whitney U comparisons test were used to compare the results among different treatment groups. Statistical significance between groups is shown by: *** p < 0.001 (MIA vs. Control), and # p < 0.05; ## p < 0.01 (MIA vs. UC groups), and $ p < 0.05; $$ p < 0.01 (pairwise comparisons between UC groups).

The articular cartilage surface was smooth, and the chondrocyte distribution was uniform in the control group in both age groups (Fig. 4a and c, respectively). As predicted, intra-articular MIA injection altered the histological structure of joint cartilage by damaging the cartilage surfaces and reducing the number of chondrocytes in the cartilage tissue. In both age groups, as shown in Fig. 4, higher doses of undenatured collagen (1.33 and 2 mg/kg) attenuated the histopathological manifestations and decreased the Mankin score compared with the MIA rats (p < 0.05 for 1.33 mg/kg; p < 0.01 for 2 mg/kg; for both age groups). Young rats administered 2 mg/kg undenatured collagen had lower Mankin scores than young rats administered 0.66 mg/kg undenatured collagen dose (p < 0.01). Old rats administered 2 mg/kg undenatured collagen had lower Mankin scores than old rats administered 0.66 (p < 0.01) and 1.33 mg/kg (p < 0.05) undenatured collagen doses.

### Serum markers

**Figure 5:**
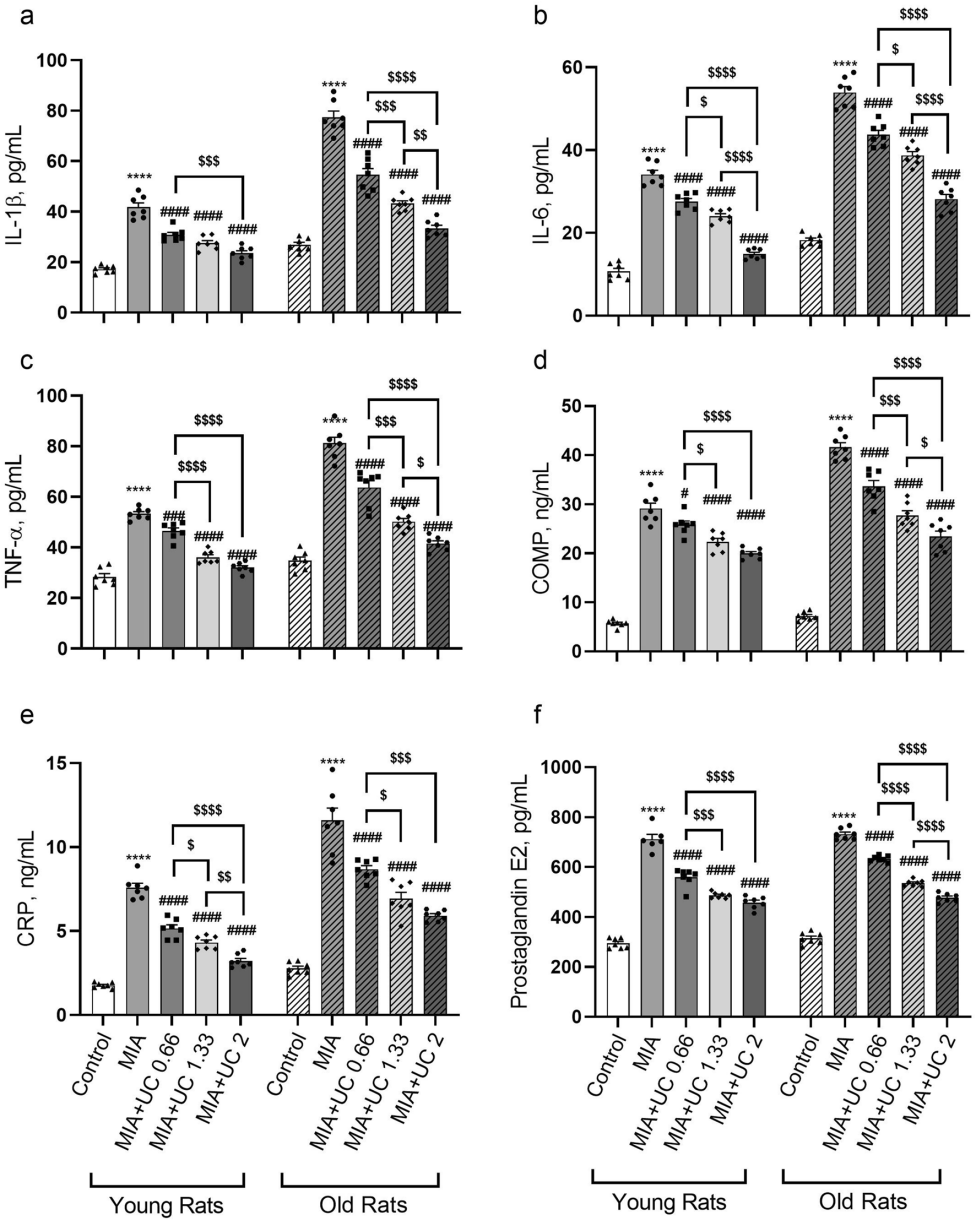
Effects of undenatured collagen (UC) supplementation at different doses on serum IL-1β (**a**), IL-6 (**b**), TNF-α (**c**), COMP (**d**), CRP (**e**), and prostaglandin E2 (**f**) levels in monosodium iodoacetate (MIA) induced osteoarthritis in young (n = 7) and old rats (n = 7). The bars represent the mean ± standard error of the mean (SEM). ANOVA and Tukey’s post hoc test were used to compare the results among different treatment groups. Statistical analysis was performed separately within each age group. Statistical significance between groups is shown by: **** p < 0.0001 (MIA vs. Control), # p < 0.05; ### p < 0.001; #### p < 0.0001 (MIA vs. UC groups), and $ p < 0.05; $$ p < 0.01; $$$ p < 0.001; $$$$ p < 0.0001 (pairwise comparisons between UC groups).

To explore the anti-inflammatory function of undenatured collagen supplementation and its effect on cartilage markers, serum levels of IL-1β, IL-6, TNF-α, COMP, CRP, and PGE2 were measured. MIA-induced OA increased all measured inflammatory markers in young and old rats (p < 0.0001, Fig. 5a–f). In young rats, all doses of undenatured collagen markedly suppressed the serum levels of IL-1β, IL-6, CRP, PGE2 (p < 0.0001, for all), TNF-α (p < 0.001 for MIA vs. MIA + UC 0.66, and p < 0.0001 for other comparisons), and COMP (p < 0.05 for MIA vs. MIA + UC 0.66 mg/kg, and p < 0.0001 for other comparisons) compared with the MIA group. The results also indicated a dose–response effect for CRP and IL-6 levels. In old rats, undenatured collagen markedly suppressed the serum IL-1β, IL-6, PGE2, TNF-α, and COMP levels in a dose-dependent manner (Fig. 5). Overall, 2 mg/kg of undenatured collagen was more effective than 0.66 mg/kg in reducing all inflammatory markers in both age groups.

### Western blot analysis

**Figure 6:**
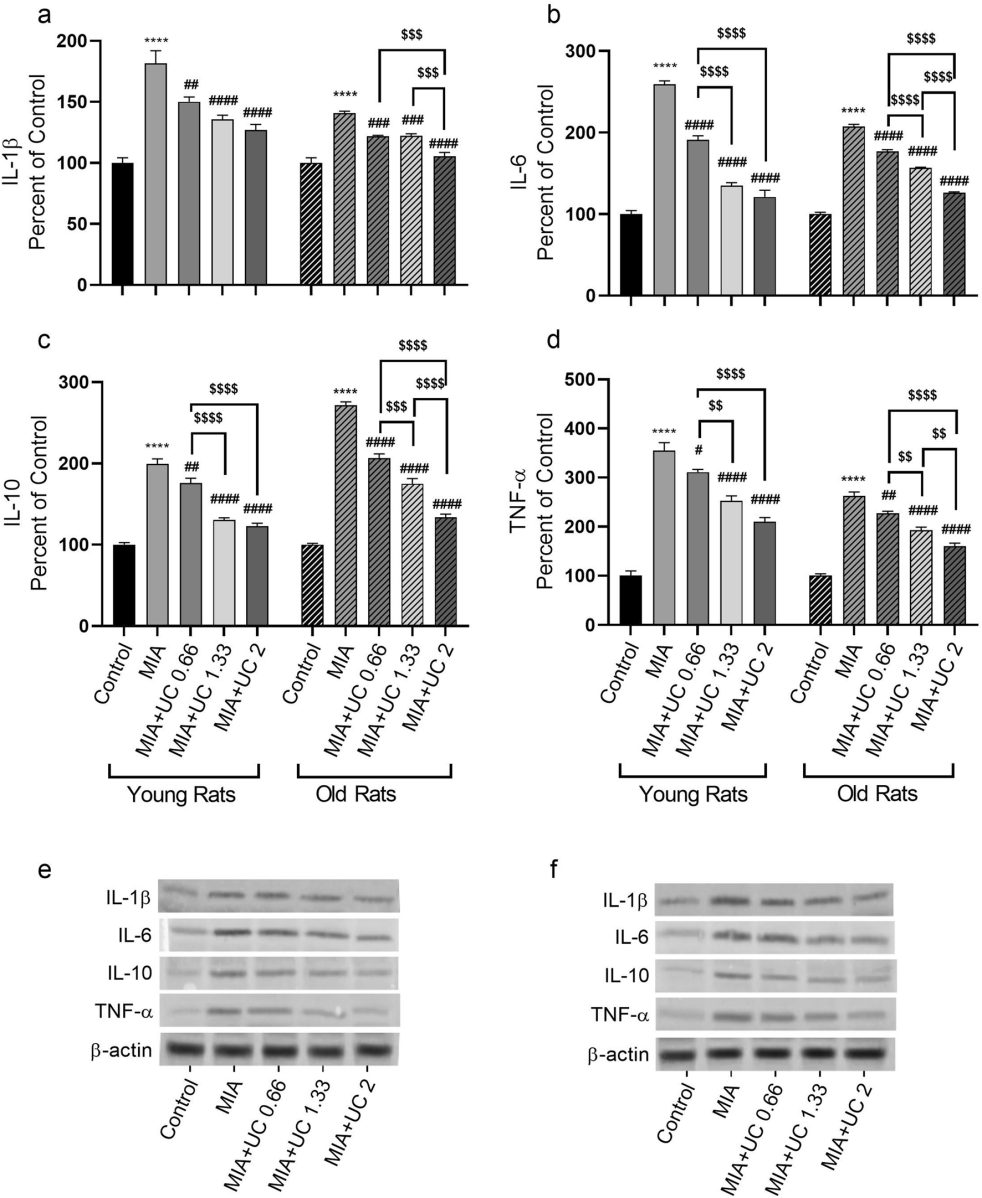
Effects of undenatured collagen (UC) supplementation at different doses on knee joint protein expression of IL-1β (**a**), IL-6 (**b**), IL-10 (**c**), and TNF-α (**d**), levels in monosodium iodoacetate (MIA) induced osteoarthritis in young and old rats. The densitometric analysis of the relative intensity according to the control group of the western blot bands was performed with β-actin normalization to ensure equal protein loading. Blots were repeated at least three times (n = 3), and representative blots were shown (**e** for young rats and **f** for old rats). The bars point out the mean ± standard error of the mean (SEM). ANOVA and Tukey’s post hoc test were used to compare the results among different treatment groups. Statistical analysis was performed separately within each age group. Statistical significance between groups is shown by: **** p < 0.0001 (MIA vs. Control), and # p < 0.05; ## p < 0.01; ### p < 0.001; #### p < 0.0001 (MIA vs. UC groups), and $$ p < 0.01; $$$ p < 0.001; $$$$ p < 0.0001 (pairwise comparisons between UC groups).

**Figure 7:**
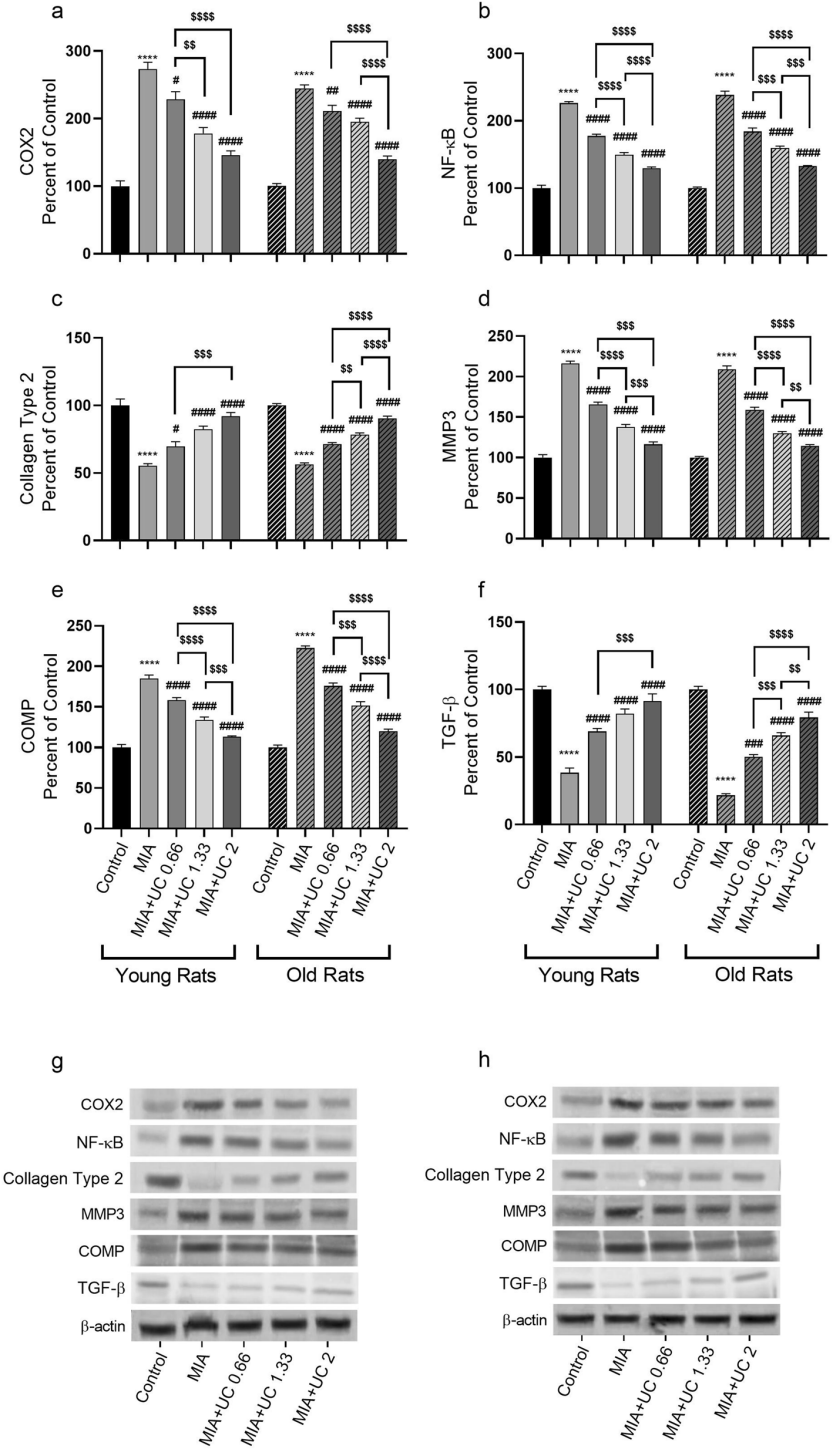
Effects of undenatured collagen (UC) supplementation at different doses on knee joint protein expression of COX2 (**a**), NF-κB (**b**), Collagen Type 2 (**c**), MMP3 (**d**), COMP (**e**), and TGF-β (**f**) levels in monosodium iodoacetate (MIA) induced osteoarthritis in young and old rats. The densitometric analysis of the relative intensity according to the control group of the western blot bands was performed with β-actin normalization to ensure equal protein loading. Blots were repeated at least three times (n = 3), and representative blots were shown (**g** for young rats and **h** for old rats). The bars point out the mean ± standard error of the mean (SEM). ANOVA and Tukey’s post hoc test were used to compare the results among different treatment groups. Statistical analysis was performed separately within each age group. Statistical significance between groups is shown by: **** p < 0.0001 (MIA vs. Control), # p < 0.05; ## p < 0.01; ### p < 0.001; #### p < 0.0001 (MIA vs. UC groups), and $$ p < 0.01; $$$ p < 0.001; $$$$ p < 0.0001(pairwise comparisons between UC groups).

MIA-induced knee OA caused inflammation as indicated by elevated expression levels of IL-1β, IL-6, IL-10, TNF-α (Fig. 6a–d), COX-2, NF-κB, MMP-3, and COMP (Fig. 7a–e) proteins in the knee joint cartilage of both young and old rats (p < 0.0001). Additionally, knee joint CTX-II and TGF-β levels were lowered in the MIA rats of both age groups (p < 0.0001). We found that undenatured collagen treatment ameliorated the expression of most inflammatory cytokines in a dose-dependent manner in both young and old rats (Fig. 6 and Fig. 7, p < 0.001). More specifically, the protein level of NF-κB, MMP3, and COMP in the knee joint was reduced in a dose-dependent manner by undenatured collagen compared with the MIA group (Fig. 7b, d and e, p< 0.001). In young rats, knee joint CTXII and TGF-β proteins were significantly improved with the 2 mg/kg dose vs. The 0.66 mg/kg dose (p < 0.001). On the other hand, in the old age group, there was a dose-dependent effect of undenatured collagen for these markers (p < 0.0001 for Collagen type 2 and p < 0.01 for TGF-β). We also found that 1.33 and 2 mg/kg undenatured collagen similarly reduced knee joint protein levels of IL-10, IL-6, TNF-α, and COX2 in young rats (p > 0.05), while in the old age group, we observed a dose-dependent effect for most of these markers.

## Discussion

Many animal^22–25^ and human^18, 26, 27^ studies have demonstrated a beneficial effect of undenatured collagen administration on OA; however, the effects of undenatured collagen in different doses and age groups have not been investigated. The present study is the first to compare the efficacy of different doses of undenatured collagen on MIA-induced OA in young and old rats. MIA injection has been shown to inhibit glyceraldehyde-3-phosphatase and lead to chondrocyte deaths^36, 37^, causing cartilage degeneration, joint space narrowing, and subchondral bone deformation in joint cartilage^38^. Radiographic and histopathological findings in response to MIA injection in both age groups agree with that reported by Orhan et al.^39^. Our data also show that intra-articular MIA injection exacerbates inflammatory status regardless of age in rats, as indicated by Ogbonna et al.^40^ and Ro et al.^17^. In addition, we demonstrated for the first time that in young and old rats, undenatured collagen (particularly 1.33 and 2 mg/kg) prevented the progression of OA by reducing markers of joint cartilage breakdown and knee swelling, improving most gait measurements, and reducing the production of inflammatory cytokines. It should be noted that previous studies have reported an oral tolerance mechanism for undenatured collagen during arthritic cartilage destruction^18,20,22,23,41^, and acute inflammatory events, in healthy subjects. For this study, we chose to focus on the effect of undenatured collagen in MIA-induced OA rats.

Based on the Kellgren-Lawrence score, MIA-injected rats had moderate OA (approximately up to grade 3), and undenatured collagen reduced OA severity (approximately to grade 1) in young rats at all doses and in old rats at 1.33 and 2 mg/kg This effect was probably linked to preventing inflammation-related subchondral bone deformation by undenatured collagen^23^. The collagen type 2-specific oral tolerance mechanism mediated by undenatured collagen can increase anti-inflammatory cytokines in joint tissue, thereby promoting the production of the articular cartilage matrix by chondrocytes^42^. Also, undenatured collagen contains cartilage matrix amino acids necessary for synthesizing and repairing connective tissue^42^. Because aging exacerbates the inflammatory state^43^ and potentially suppresses oral tolerance^44^, higher doses of undenatured collagen may be required to show better efficacy in arthritic old rats. On the other hand, inflammatory responses^43^, oral tolerance^44^, and cartilage-repairing activities can be more active in early life^45^. Therefore, in young rats, undenatured collagen administration prevented joint deformities, as reported in our previous study^23^, probably through the stimulation of oral tolerance.

Intra-articular MIA injection can increase ROS levels that cause chondrocyte death and trigger cartilage deformation, and elevate articular IL-1β, IL-6, COX2^46^, TNF-α, and NF-κB levels^23^, while reducing TGF-β levels in rats^8^. We found that undenatured collagen supplementation decreased most inflammatory cytokine activity, cartilage degradation markers (including MMP3 and COMP), and joint swelling used for joint inflammation monitoring in a dose-dependent manner. The dose-dependent functionality of undenatured collagen might have been linked to the oral tolerance mechanism that depends on age status^44^ and antigen dose^47^. The activity of oral tolerance is suppressed by aging due to the loss of functionality of Peyer’s patches and the reduction in regulatory cytokines and T-cells^48^. Consequently, the stimulability of oral tolerance may be reduced due to decreased susceptibility to oral antigen induction during aging^48^. On the other hand, a very high dose of oral antigen intake may deteriorate the oral tolerance mechanism by inactivating T-cells, while low doses stimulate the oral tolerance to produce Treg cells^49^. Our observed findings indicate that the higher doses of undenatured collagen used in this study (1.33 mg/kg and 2 mg/kg) were still below the level to deteriorate oral tolerance in both young and old rats.

COMP, a non-collagenous glycoprotein, is produced by articular chondrocytes to regulate matrix organization in cartilage^50^. The increased COMP levels in the serum and joint are considered diagnostic and prognostic OA biomarkers^51^. Similarly, MMP3, responsible for the degradation of the cartilage matrix by accelerating collagen type 2 degradation^52^, could be used as a biomarker for knee osteoarthritis^53^. The stimulation of NF-κB can exacerbate inflammatory processes and the production of joint MMP3^54^ and elevate TNF-α related serum COMP levels^55^. We showed that MIA-induced inflammation increased joint COMP and MMP3 levels regardless of age stage. The present findings also showed that undenatured collagen administration reduced NF-κB in the joint cartilage, owing to its anti-inflammatory function^23^; thus, undenatured collagen may have contributed to suppressing NF-κB-related COMP and MMP3 activity.

TGF-β plays a crucial role in the oral tolerance response by stimulating CD4+ Treg cells^56^. Undenatured collagen drives collagen type 2 specific Treg cell production in Peyer’s patches^20^. Tregs secrete anti-inflammatory cytokines, particularly TGF-β^57^, and together prevent autoimmune cartilage destruction in joint cartilage via recognizing specific self-antigens such as type 2 collagen^20^. Our findings in young rats showed that the 0.66 mg/kg and 1.33 mg/kg undenatured collagen doses similarly affected the protein expression of joint TGF-β and collagen type 2, while the 2 mg/kg dose showed the best efficacy. This could be attributed to the oral tolerance mechanism being more active in early life^58^. TGF-β stimulatory activity of undenatured collagen-stimulated Treg cells may have partially contributed to chondrocytes survival for cartilage repair^59^. Aging decreases anabolic factors and stimulates catabolic and inflammatory activity in chondrocytes^60^. This age-related catabolic activity probably led to a dose-dependent alteration impacting TGF-β and collagen type 2 levels in old rats. TGF-β is a key regulator of collagen type 2 expression in chondrocytes, but aging may dampen this regulatory activity^59^ because chondrocytes become less sensitive to TGF-β-stimulated proliferative activity^60^. Taken together, we can assume that the efficiency of the same dose of undenatured collagen may decline with age due to the loss of oral tolerance functionality^44^.

Irregularity in the articular surface is one of the main findings of OA that originated from the degradation of the collagen matrix and cartilage damage^19^. The anti-inflammatory and collagen type 2 stimulating activity of undenatured collagen may help cartilage regeneration against MIA-induced OA^22, 23^. Bagi et al.^24^ demonstrated that a low dose of 40 mg HED of undenatured collagen for 8 weeks could prevent cartilage damage in rats in a different OA model induced by partial medial meniscectomy tear. In the present study, according to Mankin scores, only 1.33 and 2 mg/kg undenatured collagen (equivalent to 80 and 120 mg HED) administration attenuated cartilage degradation in young and old rats. The difference between Bagi et al.^24^ and our study may have stemmed from the OA induction method and the longer duration of undenatured collagen administration. Previously we reported that a 40 mg HED dose could not reduce the Mankin score in MIA rats^23^ as in the present study. This could be because higher doses of undenatured collagen were required to alleviate cartilage damage by regenerating the cartilage microstructure in rats.

Previous studies show that OA treatment using undenatured collagen alleviates joint pain and improves quality of life^27^. Although a low dose of undenatured collagen did not effectively attenuate joint cartilage damage in old rats, the present findings show that all doses of undenatured collagen supplementation reduced knee swelling and improved gait patterns, closely related to pressure pain thresholds. These results are probably related to decreased pain sensitivity and increased pain threshold parallel to ageig^61^.

Supplementation with the different doses of undenatured collagen used in this study appears to be safe. We did not observe any changes in body weight, serum glucose, BUN, creatine, ALT, and AST levels in young or old rats (Supplementary Table 1). The MIA-induced OA model is less severe regarding the pain phenotype and adverse effects on animal welfare^62^. Therefore, there was no worsening of body conditions due to the MIA injection, and knee joint swelling severity was not enough to affect the well-being of MIA rats. In addition, we did not detect any pathomorphological, hematological, or biochemical changes after different doses of undenatured collagen. Similar observations with 0.66 mg/kg of undenatured collagen have beenreported in previous studies^22, 23, 63, 64^.

This study presents several limitations. First, the influence of hormonal effect during aging needed to be considered, especially since estrogen levels regulate changes in OA by preventing degradation of the extracellular matrix^65^. In this study, we were unable to evaluate the difference in the development of osteoarthritis caused by varied estrogen levels in young and aged rats. In addition, the effect of anti-inflammatory and joint health-promoting activity of undenatured collagen on healthy animals should be clarified. Lastly, the exact mechanism of action of undenatured collagen in OA is still lacking, and additional mechanistic studies are needed to achieve greater understanding of the ingredient.

## Conclusion

In the current study, we showed for the first time the activity of various doses of undenatured collagen in reducing OA symptoms in young and old rats. We concluded that while the lower dose of undenatured collagen (0.66 mg/kg) partially ameliorates MIA-induced OA symptoms, and protects the joint structure in rats, higher doses work faster and are more efficacious in improving overall joint health. Also, this preliminary data indicated that lower doses of undenatured collagen might help treat either injury- or age-related OA in different age categories in rats. However, there is a need for comprehensive clinical studies in humans or animals with OA to support our present findings.

## Supplementary Information

Supplementary Information.

## Data Availability

Correspondence and requests for materials should be addressed to K. Sahin.

## References

[1] Hunter DJ, March L, Chew M (2020). Osteoarthritis in 2020 and beyond: A Lancet commission. Lancet.

[2] Deshpande BR (2016). Number of persons with symptomatic knee osteoarthritis in the us: Impact of race and ethnicity, age, sex, and obesity. Arthritis Care. Res. (Hobok.).

[3] Ackerman IN, Kemp JL, Crossley KM, Culvenor AG, Hinman RS (2017). Hip and knee osteoarthritis affects younger people, too. J. Orthop. Sports. Phys. Ther..

[4] Driban JB, Harkey MS, Liu S-H, Salzler M, McAlindon TE (2020). Osteoarthritis and aging: Young adults with osteoarthritis. Curr. Epidemiol. Rep..

[5] Loeser RF, Collins JA, Diekman BO (2016). Ageing and the pathogenesis of osteoarthritis. Nat. Rev. Rheumatol..

[6] Greene MA, Loeser RF (2015). Aging-related inflammation in osteoarthritis. Osteoarthr. Cartil..

[7] Rigoglou S, Papavassiliou AG (2013). The NF-κB signalling pathway in osteoarthritis. Int. J. Biochem. Cell Biol..

[8] Korotkyi O (2020). Cytokines profile in knee cartilage of rats during monoiodoacetate-induced osteoarthritis and administration of probiotic. Biopolym. Cell.

[9] Houard X, Goldring MB, Berenbaum F (2013). Homeostatic mechanisms in articular cartilage and role of inflammation in osteoarthritis. Curr. Rheumatol. Rep..

[10] Lu J, Zhang T, Sun H, Wang S, Liu M (2018). Protective effects of dioscin against cartilage destruction in a monosodium iodoacetate (MIA)-indcued osteoarthritis rat model. Biomed. Pharmacother..

[11] Korotkyi O (2020). The gut microbiota of rats under experimental osteoarthritis and administration of chondroitin sulfate and probiotic. Mikrobiol. Z..

[12] Wu Y-L (2021). Gut microbiota alterations and health status in aging adults: From correlation to causation. Aging Med..

[13] Hsia AW (2021). Post-traumatic osteoarthritis progression is diminished by early mechanical unloading and anti-inflammatory treatment in mice. Osteoarthr. Cartil..

[14] Heijink A (2012). Biomechanical considerations in the pathogenesis of osteoarthritis of the knee. Knee Surg. Sports Traumatol. Arthrosc..

[15] Mapp PI (2013). Differences in structural and pain phenotypes in the sodium monoiodoacetate and meniscal transection models of osteoarthritis. Osteoarthr. Cartil..

[16] de Sousa Valente J (2019). The pharmacology of pain associated with the monoiodoacetate model of osteoarthritis. Front. Pharmacol..

[17] Ro JY (2020). Age and sex differences in acute and osteoarthritis-like pain responses in rats. J. Gerontol. A Biol. Sci. Med. Sci..

[18] Bagchi D (2002). Effects of orally administered undenatured type II collagen against arthritic inflammatory diseases: Va mechanistic exploration. Int. J. Clin. Pharmacol. Res..

[19] Harris RB, Fonseca FLA, Sharp MH, Ottinger CR (2022). Functional characterization of undenatured type II collagen supplements: Are they interchangeable?. J. Diet. Suppl..

[20] Gencoglu H, Orhan C, Sahin E, Sahin K (2020). Undenatured type II collagen (UC-II) in joint health and disease: A review on the current knowledge of companion animals. Animals.

[21] Asnagli H (2014). Type 1 regulatory T cells specific for collagen type II as an efficient cell-based therapy in arthritis. Arthritis Res. Ther..

[22] Sahin K (2021). Niacinamide and undenatured type II collagen modulates the inflammatory response in rats with monoiodoacetate-induced osteoarthritis. Sci. Rep..

[23] Orhan C (2021). Undenatured type ii collagen ameliorates inflammatory responses and articular cartilage damage in the rat model of osteoarthritis. Front. Vet. Sci..

[24] Bagi CM, Berryman ER, Teo S, Lane NE (2017). Oral administration of undenatured native chicken type II collagen (UC-II) diminished deterioration of articular cartilage in a rat model of osteoarthritis (OA). Osteoarthr. Cartil..

[25] Varney JL, Fowler JW, Coon CN (2021). Undenatured type II collagen mitigates inflammation and cartilage degeneration in healthy Labrador Retrievers during an exercise regimen. Transl. Anim. Sci..

[26] Costa A (2020). Associated strengthening exercises to undenatured oral type II collagen (UC-II). A randomized study in patients affected by knee osteoarthritis. Muscles Ligaments Tendons J..

[27] Sadigursky D (2022). Undenatured collagen type II for the treatment of osteoarthritis of the knee. Acta. Ortop. Bras..

[28] Di-Cesare-Mannelli L, Micheli L, Zanardelli M, Ghelardini C (2013). Low dose native type II collagen prevents pain in a rat osteoarthritis model. BMC Musculoskelet. Disord..

[29] Gupta RC (2009). Therapeutic efficacy of undenatured type-II collagen (UC-II) in comparison to glucosamine and chondroitin in arthritic horses1. J. Vet. Pharmacol. Ther..

[30] Ganesan K (2008). Gender differences and protective effects of testosterone in collagen induced arthritis in rats. Rheumatol. Int..

[31] Lugo JP, Saiyed ZM, Lane NE (2016). Efficacy and tolerability of an undenatured type II collagen supplement in modulating knee osteoarthritis symptoms: A multicenter randomized, double-blind, placebo-controlled study. Nutr. J..

[32] Shin J-W, Seol I-C, Son C-G (2010). Interpretation of animal dose and human equivalent dose for drug development. J. Korean. Med. Sci..

[33] Aravinthan A (2021). Ginsenoside Rb1 inhibits monoiodoacetate-induced osteoarthritis in postmenopausal rats through prevention of cartilage degradation. J. Ginseng. Res..

[34] Kellgren JH, Lawrence JS (1957). Radiological assessment of osteo-arthrosis. Ann. Rheum..

[35] Mankin HJ, Dorfman H, Lippiello L, Zarins A (1971). Biochemical and metabolic abnormalities in articular cartilage from osteo-arthritic human hips. II. Correlation of morphology with biochemical and metabolic data. J. Bone Joint Surg. Am..

[36] Sabri MI, Ochs S (1971). Inhibition of glyceraldehyde-3-phosphate Dehydrogenase in mammalian nerve by iodoacetic acid. J. Neurochem..

[37] Stevenson GW (2011). Monosodium iodoacetate-induced osteoarthritis produces pain-depressed wheel running in rats: Implications for preclinical behavioral assessment of chronic pain. Pharmacol. Biochem. Behav..

[38] Pitcher T, Sousa-Valente J, Malcangio M (2016). The monoiodoacetate model of osteoarthritis pain in the mouse. J. Vis. Exp..

[39] Orhan C (2022). Protective effect of a novel polyherbal formulation on experimentally induced osteoarthritis in a rat model. Biomed. Pharmacother..

[40] Ogbonna AC, Clark AK, Malcangio M (2015). Development of monosodium acetate-induced osteoarthritis and inflammatory pain in ageing mice. Age.

[41] Deparle LA, Gupta RC, Canerdy TD, Goad JT, D’Altilio M, Bagchi M, Bagchi D (2005). Efficacy and safety of glycosylated undenatured type II collagen (UC-II) in therapy of arthritic dogs. J. Vet. Pharmacol. Therap..

[42] Sadigursky D, Sanches MM, Garcia NM, Cantão MDO, Matos MA (2023). Effectiveness of the use of non-hydrolysed type II collagen in the treatment of osteoarthritis: A systematic review and meta-analysis. Braz. J. Heath Rev..

[43] Kale SS, Yende S (2011). Effects of aging on inflammation and hemostasis through the continuum of critical illness. Aging. Dis..

[44] Kato H (2003). Lack of oral tolerance in aging is due to sequential loss of Peyer’s patch cell interactions. Int. Immunol..

[45] Wilson B, Novakofski KD, Donocoff RS, Liang YX, Fortier LA (2014). Telomerase activity in articular chondrocytes is lost after puberty. Cartilage.

[46] Moilanen LJ (2015). Monosodium iodoacetate-induced inflammation and joint pain are reduced in TRPA1 deficient mice & #x2013; potential role of TRPA1 in osteoarthritis. Osteoarthr. Cartil..

[47] Spahn TW (2002). Mesenteric lymph nodes are critical for the induction of high-dose oral tolerance in the absence of Peyer's patches. Eur. J. Immunol..

[48] Santiago AF (2011). Aging correlates with reduction in regulatory-type cytokines and T cells in the gut mucosa. Immunobiology.

[49] Weiner HL, da Cunha AP, Quintana F, Wu H (2011). Oral tolerance. Immunol. Rev..

[50] Tseng S, Reddi AH, Di Cesare PE (2009). Cartilage oligomeric matrix protein (COMP): A biomarker of arthritis. Biomark. Insights.

[51] Verma P, Dalal K (2013). Serum cartilage oligomeric matrix protein (COMP) in knee osteoarthritis: A novel diagnostic and prognostic biomarker. J. Orthop. Res..

[52] Chen J-J, Huang J-F, Du W-X, Tong P-J (2014). Expression and significance of MMP3 in synovium of knee joint at different stage in osteoarthritis patients. Asian Pac. J. Trop. Med.

[53] Pengas I (2018). MMP-3 in the peripheral serum as a biomarker of knee osteoarthritis, 40 years after open total knee meniscectomy. J. Exp. Orthop..

[54] Sun C (2022). A2M inhibits inflammatory mediators of chondrocytes by blocking IL-1β/NF-κB pathway. J. Orthop. Res..

[55] Happonen KE (2012). Serum COMP-C3b complexes in rheumatic diseases and relation to anti-TNF-α treatment. Arthritis Res. Ther..

[56] Rezende RM, Cox LM, Weiner HL (2019). Mucosal tolerance therapy in humans: Past and future. Clin. Exp. Neuroimmunol..

[57] Wan YY, Flavell RA (2007). 'Yin-Yang' functions of transforming growth factor-beta and T regulatory cells in immune regulation. Immunol. Rev..

[58] Yokanovich LT, Newberry RD, Knoop KA (2021). Regulation of oral antigen delivery early in life: Implications for oral tolerance and food allergy. Clin. Exp. Allergy.

[59] Zhu Y (2015). Transforming growth factor-β1 induces type II collagen and aggrecan expression via activation of extracellular signal-regulated kinase 1/2 and Smad2/3 signaling pathways. Mol. Med. Rep..

[60] Lotz M, Loeser RF (2012). Effects of aging on articular cartilage homeostasis. Bone.

[61] Lautenbacher S, Peters JH, Heesen M, Scheel J, Kunz M (2017). Age changes in pain perception: A systematic-review and meta-analysis of age effects on pain and tolerance thresholds. Neurosci. Biobehav. Rev..

[62] Bove SE (2003). Weight bearing as a measure of disease progression and efficacy of anti-inflammatory compounds in a model of monosodium iodoacetate-induced osteoarthritis. Osteoarthr. Cartil..

[63] Orhan C (2021). Effects of exercise combined with undenatured type II collagen on endurance capacity, antioxidant status, muscle lipogenic genes and e3 ubiquitin ligases in rats. Animals.

[64] Marone PA, Lau FC, Gupta RC, Bagchi M, Bagchi D (2010). Safety and toxicological evaluation of undenatured type II collagen. Toxicol. Mech. Methods..

[65] Xu X (2019). Estrogen modulates cartilage and subchondral bone remodeling in an ovariectomized rat model of postmenopausal osteoarthritis. Med. Sci. Monit..

