# Supplementary material for: The effect of oral administration of undenatured type II collagen on monosodium iodoacetate-induced osteoarthritis in young and old rats
Source: Sci Rep. 2023 Apr 20;13:6499. doi: 10.1038/s41598-023-33763-2 (PMC10119188; doi:10.1038/s41598-023-33763-2)
Supplement: Supplementary file 1 — Supplementary Information. [file 41598_2023_33763_MOESM1_ESM.docx]

**The effect of oral administration of undenatured type II collagen on monosodium iodoacetate-induced osteoarthritis in young and old rats**

**Emre Sahin^1^, Cemal Orhan^2^, Fusun Erten^3^, Zainulabedin Saiyed^4^, Elnaz Karimian Azari^4^, Shane Durkee^4^, Kazim Sahin^2^***

1Department of Animal Nutrition, Faculty of Veterinary Medicine, Bingol University, Bingol, 12100, Turkey

2Department of Animal Nutrition, Faculty of Veterinary Medicine, Firat University, Elazig, 23119, Turkey

3Department of Veterinary Science, Pertek Sakine Genc Vocational School, Munzur University, Tunceli, 62500 Turkey

4Lonza Greenwood LLC, Greenwood, NC, 6024, USA

* nsahinkm@yahoo.com

# Material and methods

**Safety Markers**

An automatic biochemistry analyzer was used to measure glucose, blood urine nitrogen (BUN), creatinine, alanine aminotransferase (ALT), and aspartate aminotransferase (AST) levels in the serum (Samsung LABGEOPT10, Suwon, Korea), using rat specific commercial kits.

**Results**

**Body Weight and Safety Markers**

Intra-articular MIA injection or different doses of undenatured collagen administration did not significantly alter final body weight, serum glucose, BUN, creatine, ALT, and AST levels in old and young rats (p>0.05, Supplementary Table 1).

## Supplementary Tables

**Table S1.** Effects of undenatured collagen (UC) supplementation at different doses on final body weight and serum biochemical parameters in monosodium iodoacetate (MIA) induced osteoarthritis in young and old rats.

| Items | | Groups | | | | | *--P--** |
| --- | --- | --- | --- | --- | --- | --- | --- |
|  |  | Control | MIA | MIA+UC 0.66 | MIA+UC 1.33 | MIA+UC 2.00 |  |
| Final body weight, g | Young | 298.17±13.44 | 294.40±12.17 | 295.43±9.65 | 295.67±12.92 | 297.00±9.34 | 0.999 |
|  | Old | 340.71±7.97 | 337.71±6.99 | 338.43±6.11 | 340.00±7.53 | 340.33±6.00 | 0.998 |
| Glucose, mg/dL | Young | 114.71±2.39 | 115.57±3.04 | 116.00±2.33 | 115.71±2.66 | 116.29±3.03 | 0.995 |
|  | Old | 114.43±1.69 | 116.43±3.33 | 115.14±2.78 | 116.14±5.34 | 114.57±2.79 | 0.990 |
| BUN, g/dL | Young | 20.07±0.28 | 20.41±0.56 | 20.74±0.79 | 20.89±0.71 | 20.21±0.73 | 0.882 |
|  | Old | 21.57±0.45 | 21.11±0.61 | 21.67±0.47 | 21.10±0.68 | 21.04±0.34 | 0.860 |
| Creatine, mg/dL | Young | 0.37±0.02 | 0.36±0.02 | 0.37±0.02 | 0.36±0.03 | 0.37±0.03 | 0.997 |
|  | Old | 0.46±0.02 | 0.50±0.01 | 0.48±0.02 | 0.49±0.03 | 0.46±0.01 | 0.337 |
| ALT, U/L | Young | 109.71±4.14 | 111.86±2.99 | 109.29±4.26 | 107.86±3.02 | 110.29±7.17 | 0.981 |
|  | Old | 118.57±3.85 | 117.71±5.47 | 115.00±5.91 | 120.43±3.80 | 117.29±4.72 | 0.953 |
| AST, U/L | Young | 117.86±4.62 | 120.57±4.08 | 119.86±4.45 | 121.29±5.18 | 118.86±6.51 | 0.990 |
|  | Old | 129.71±4.52 | 134.70±5.19 | 130.69±7.07 | 133.69±5.93 | 131.77±4.54 | 0.966 |
| BUN: Blood urea nitrogen; ALT: Alanine aminotransferase; AST: Aspartate aminotransferase. *One-way ANOVA test. Mean values of items are demonstrated with ± standard error of mean. | | | | | | | |

**Supplementary Figures**

## Supplementary Figure 1

A) Young Rats IL-1β


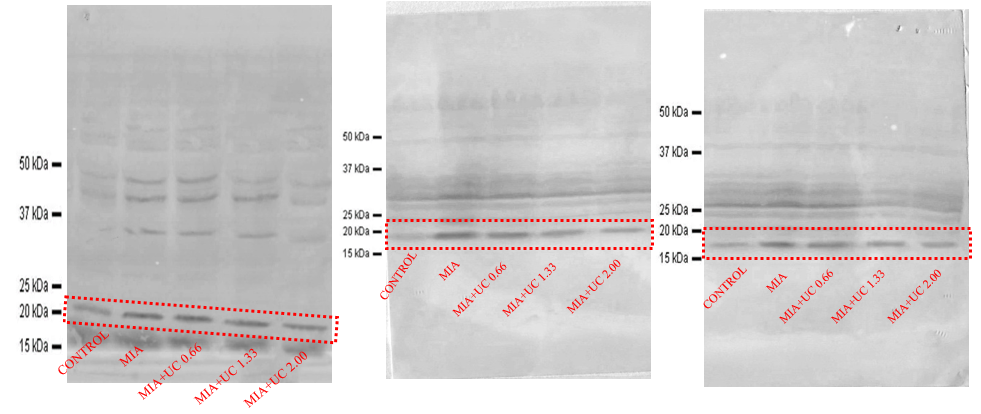


B) Young Rats IL-6


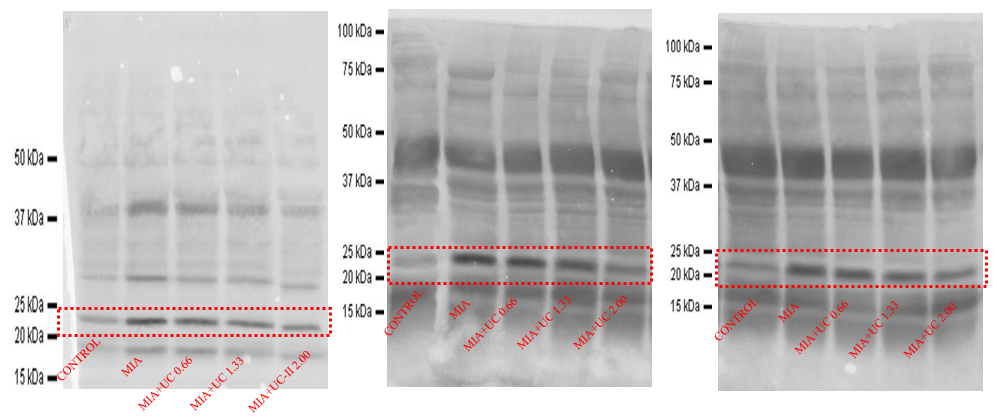


C) Young Rats IL-10


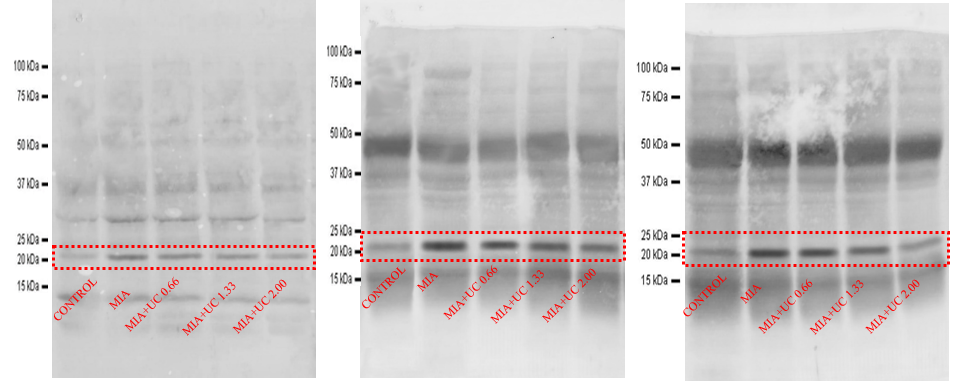


D) Young Rats TNF-α


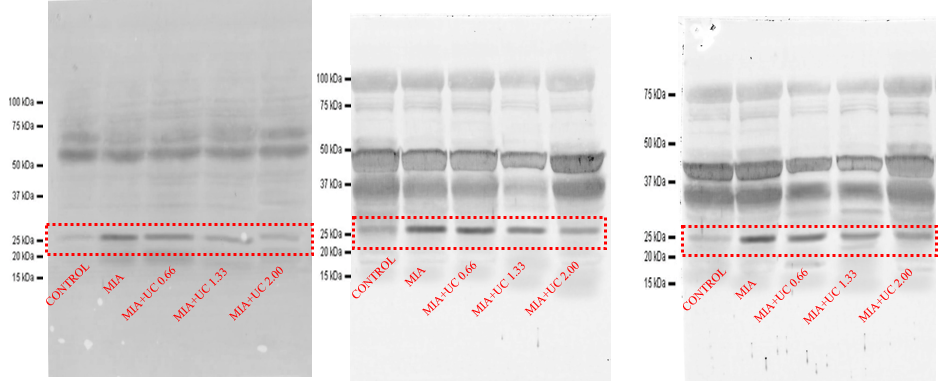


E) Young Rats β-actin


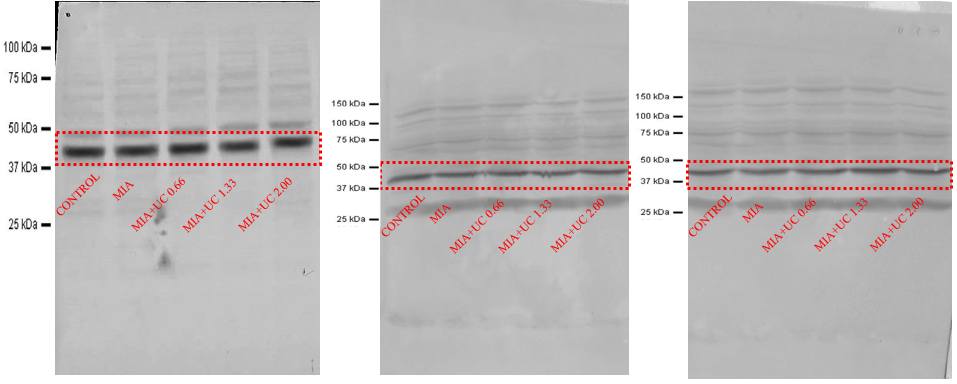


F) Old Rats IL-1β


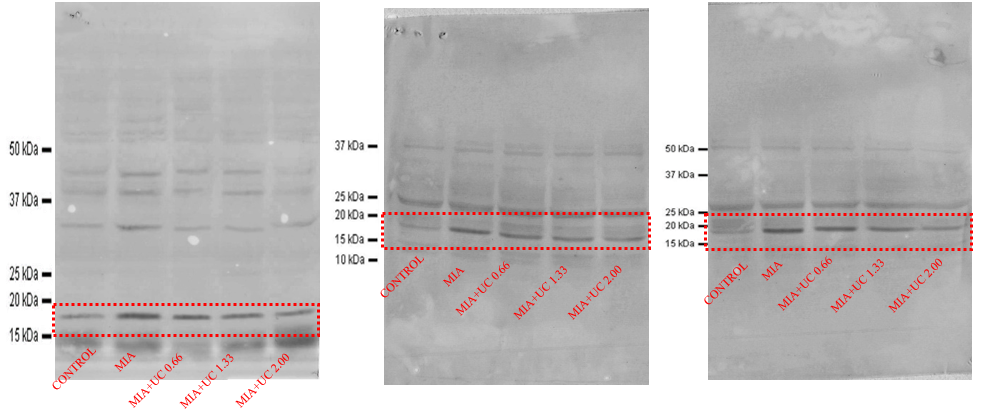


G) Old Rats IL-6


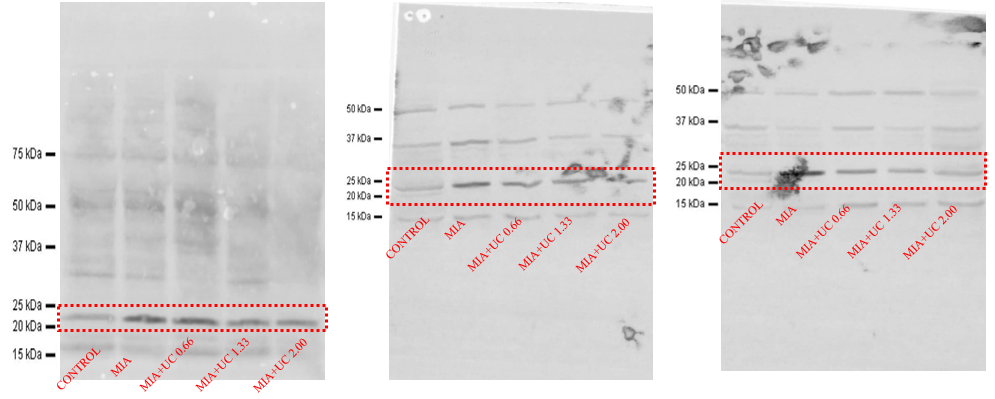


H) Old Rats IL-10


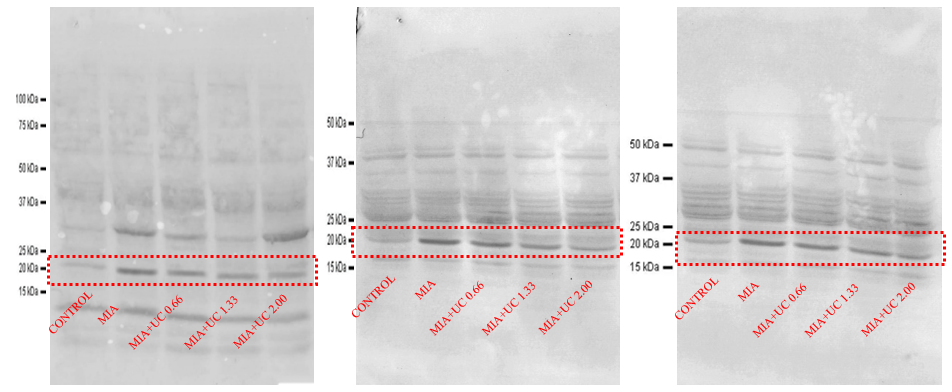


I) Old Rats TNF-α


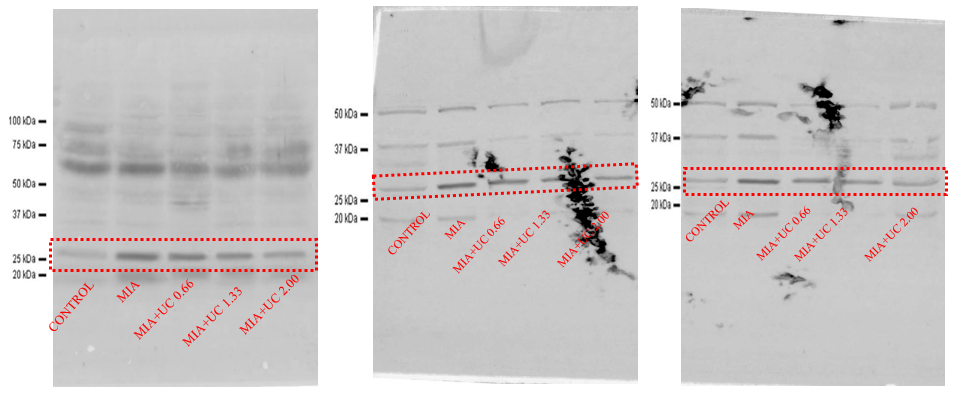


J) Old Rats β-actin


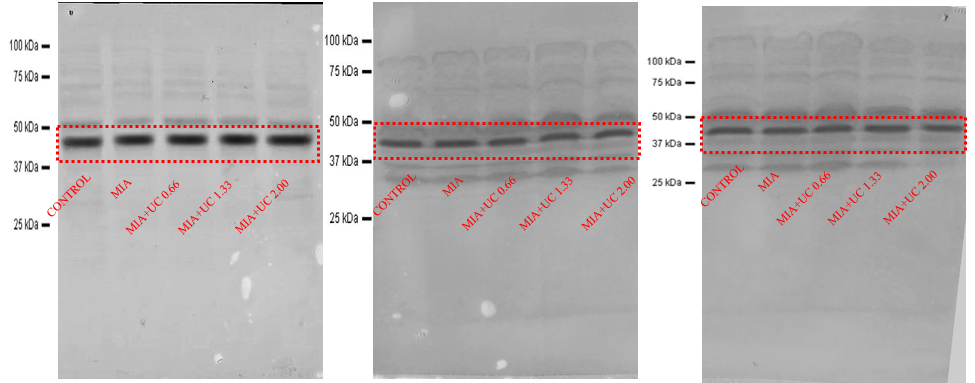


**Figure S1.** Full immunoblots related to Figure 5 in knee joint tissue of young rats; IL-1β (A), IL-6 (B), IL-10 (C), TNF-α (D), and β-actin (E) Figure 5 on in knee joint tissue of old rats; IL-1β (F), IL-6 (G), IL-10 (H), TNF-α (I), and β-actin (J). Each immunoblot is a representative of three independent experiments. Results shown in Fig 5. are delineated by red dotted rectangles. MW (in kDa) are indicated. IL-1β, Interleukin 1 beta; IL-6, Interleukin 6; IL-10, Interleukin-10; TNF-α, Tumor necrosis factor-alpha. Since the analysis of different studies was performed in the same gel medium, the bands belonging to this study are presented as they are. In the study, nitrocellulose membranes were cut at the blotting stage and different antibody applications were made.

## Supplementary Figure 2

A) Young Rats COX2


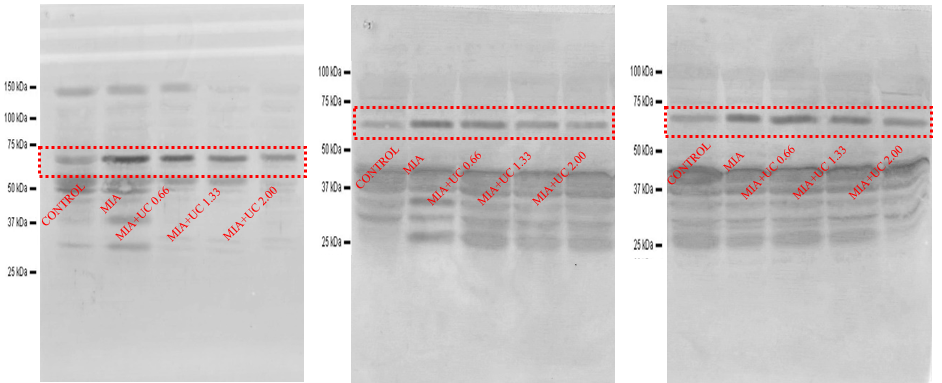


B) Young Rats NF-κB


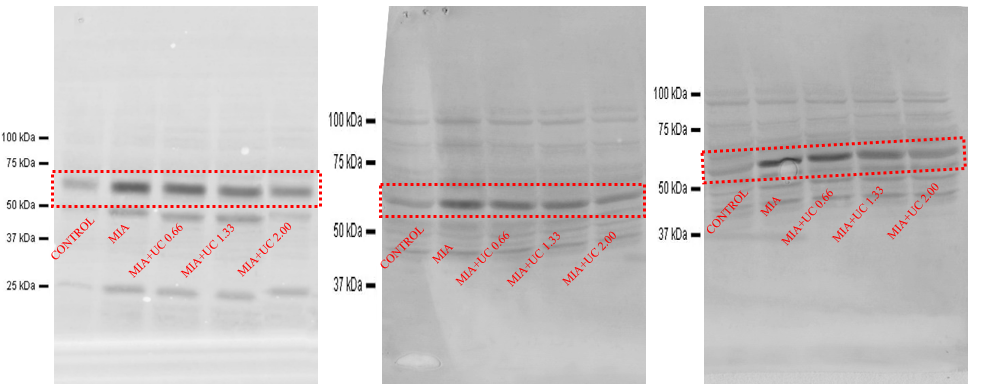


C) Young Rats Collagen Type 2


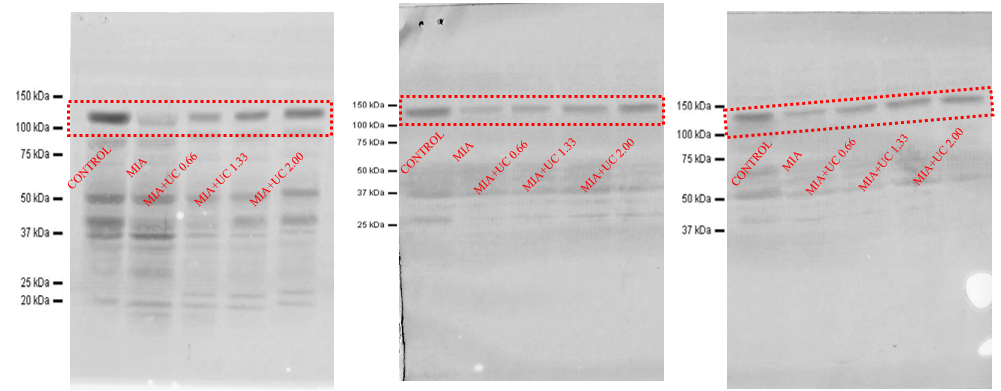


D) Young Rats MMP3


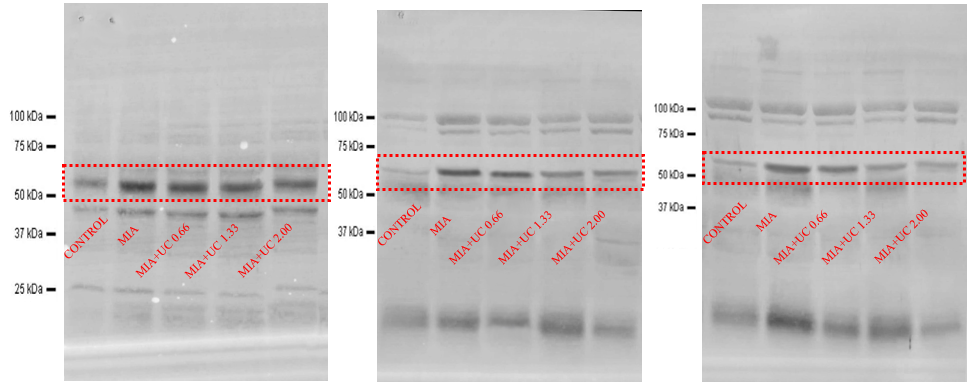


E) Young Rats COMP


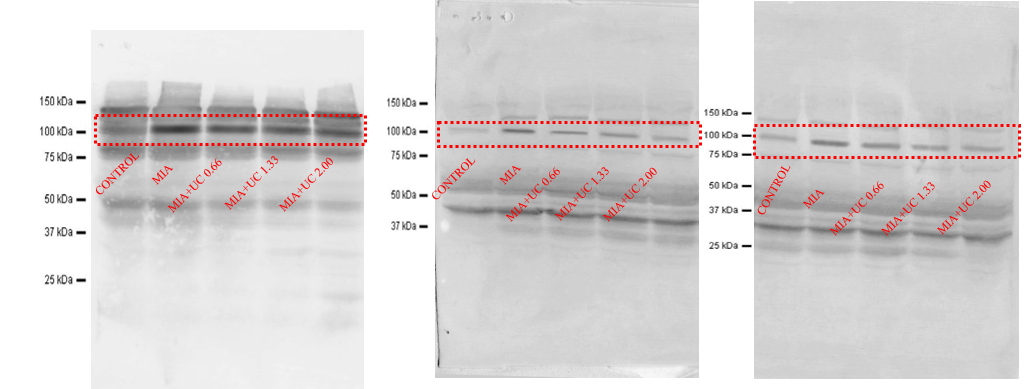


F) Young Rats TGF-β


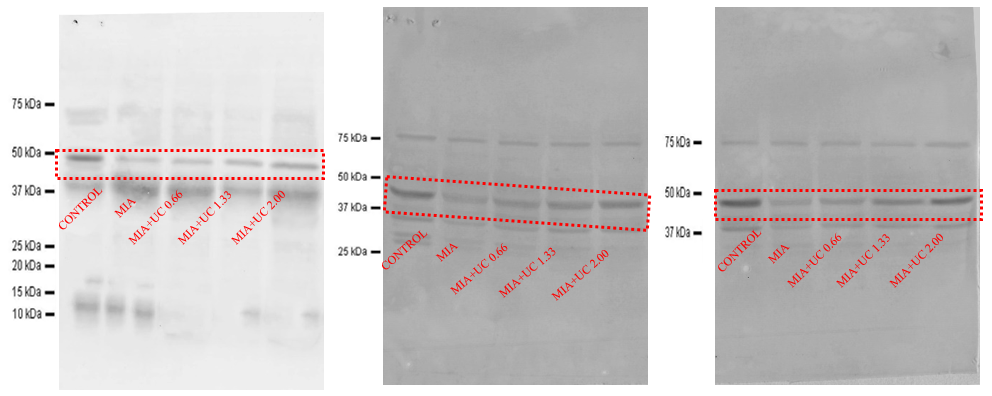


G) Young Rats β-actin


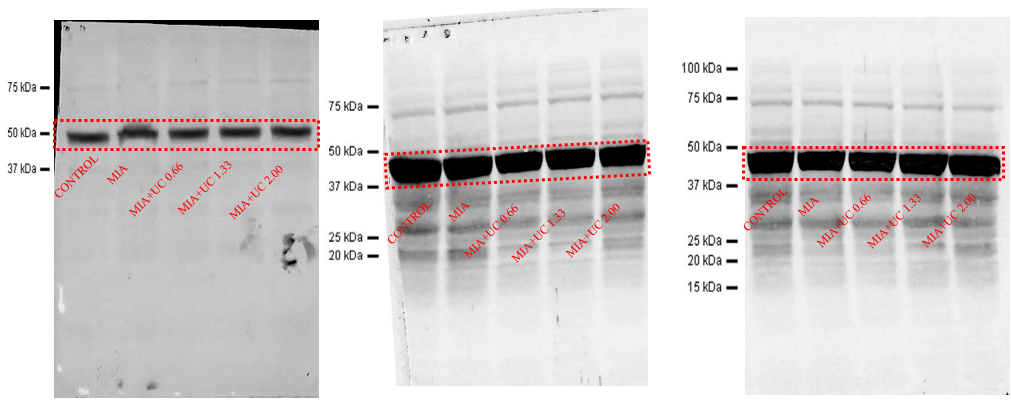


H) Old Rats COX2


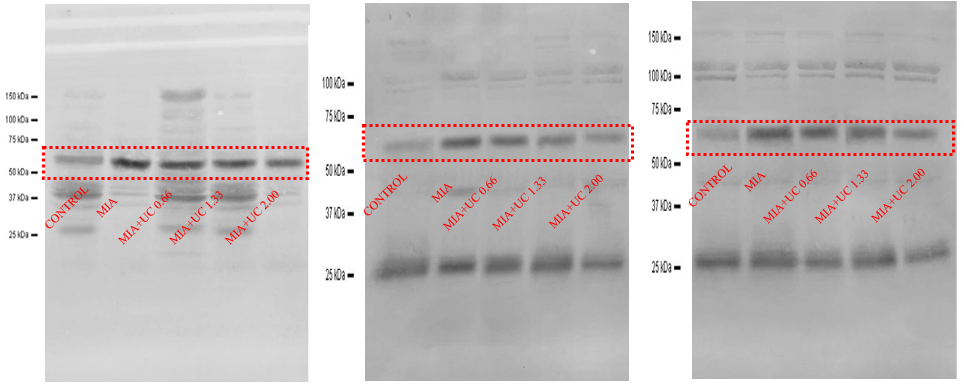


I) Old Rats NF-ĸB


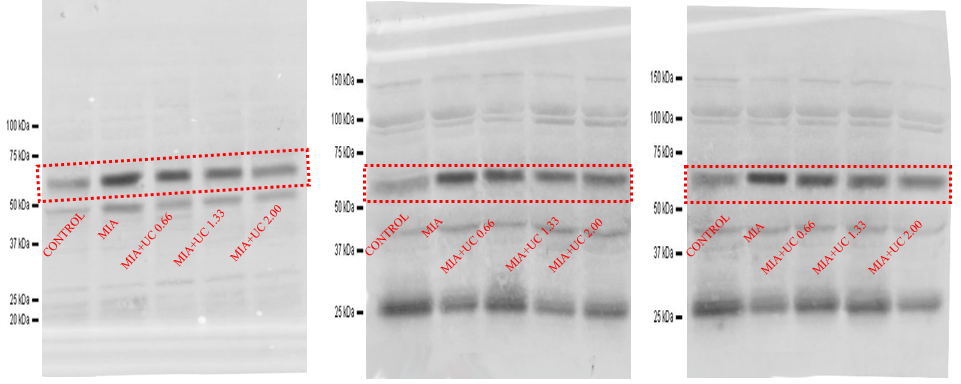


J) Old Rats Collagen Type 2


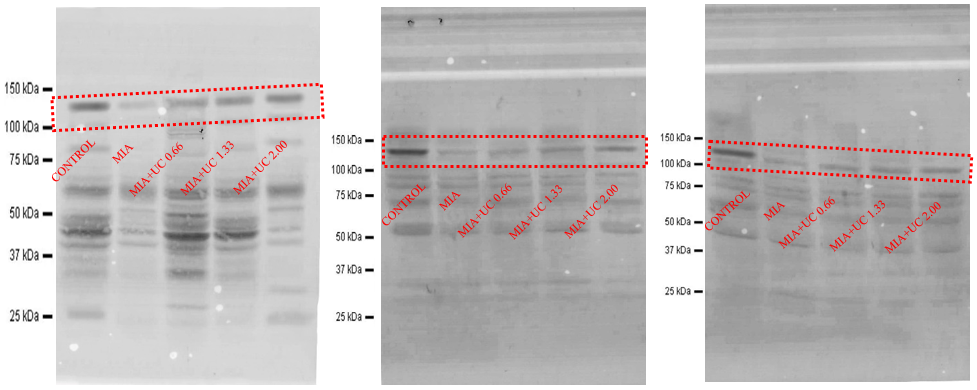


K) Old Rats MMP3


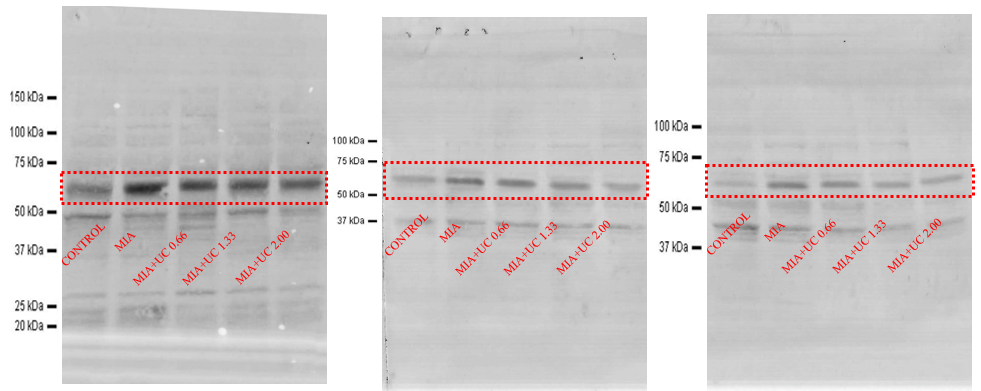


L) Old Rats COMP


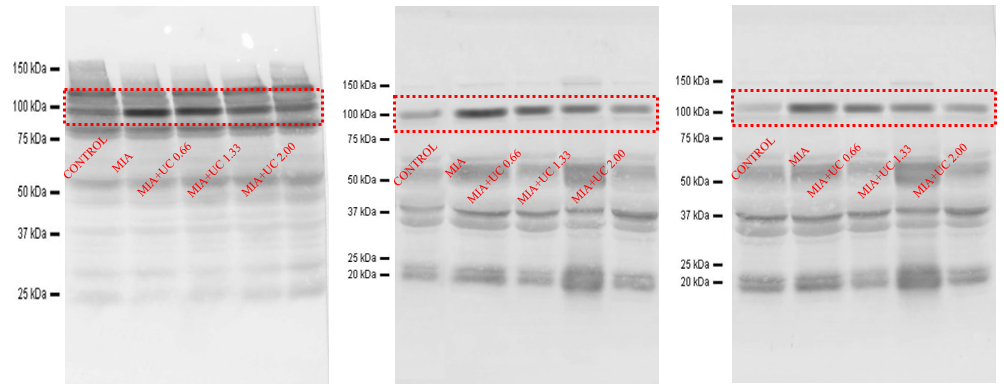


M) Old Rats TGF-β


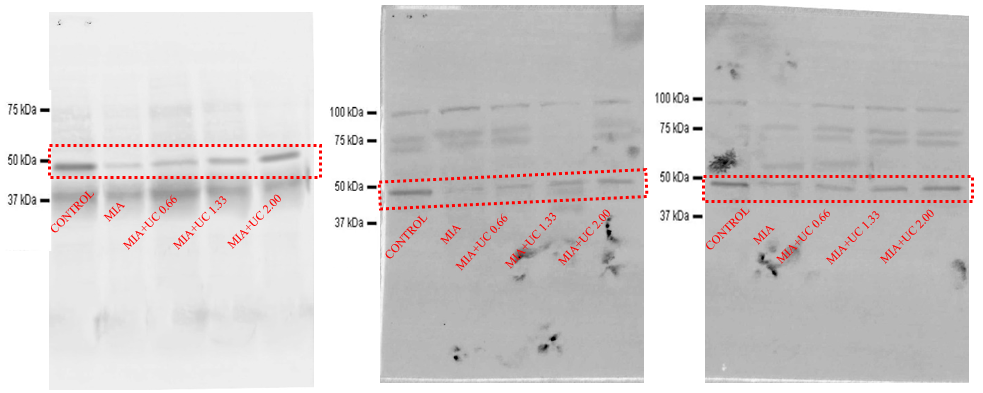


N) Old Rats β-actin


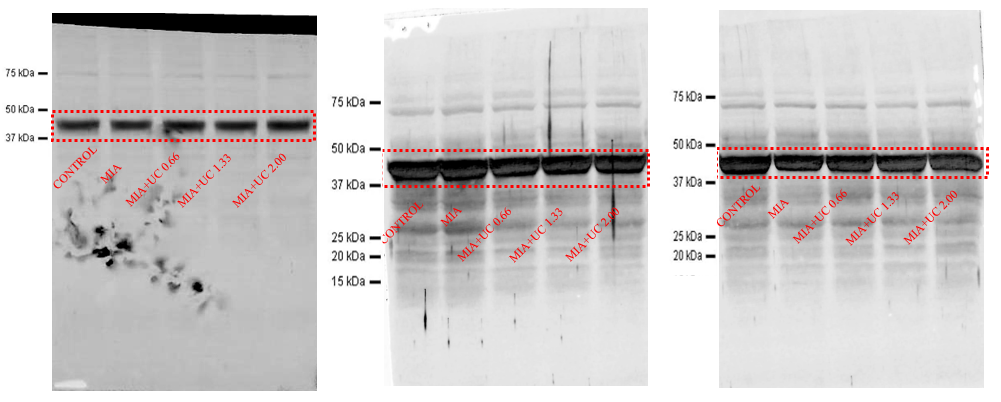


**Figure S2.** Full immunoblots related to Figure 6 on knee joint protein tissue of young rats; COX2 (A), NF-κB (B), Collagen Type 2 (C), MMP3 (D), COMP (E), TGF-β (F), and β-actin (G). Fig. 6 on knee joint tissue of old rats; COX2 (H), NF-κB (I), Collagen Type 2 (J), MMP3 (K), COMP (L), TGF-β (M), and β-actin (N). Each immunoblot is a representative of three independent experiments. Results shown in Fig. 6 are delineated by red dotted rectangles. MW (in kDa) are indicated. COX-2, cyclooxygenase-2; NF-κB, Nuclear Factor kappa B; MMP-3, matrix metalloproteinase-3; COMP, Cartilage oligomeric matrix protein; TGF-β, Transforming growth factor-β. Since the analysis of different studies was performed in the same gel medium, the bands belonging to this study are presented as they are. In the study, nitrocellulose membranes were cut at the blotting stage and different antibody applications were made.
